# Effect of Metal Bionanohybrids on Pre-formed Marine Biofilms

**DOI:** 10.1021/acsabm.5c01575

**Published:** 2025-10-03

**Authors:** Clara Ortega-Nieto, Maria J. Romeu, Rita Teixeira-Santos, Luciana C. Gomes, Filipe J. Mergulhão, Jose M. Palomo

**Affiliations:** † 16379 Instituto de Catalisis y Petroleoquimica (ICP), CSIC, C/Marie Curie 2, Madrid 28049 Spain; ‡ LEPABE - Laboratory for Process Engineering, Environment, Biotechnology and Energy, Faculty of Engineering 386292 University of Porto, Rua Dr. Roberto Frias, Porto 4200-465, Portugal; § ALiCE - Associate Laboratory in Chemical Engineering, Faculty of Engineering, University of Porto, Rua Dr. Roberto Frias, Porto 4200-465, Portugal

**Keywords:** bacterial biofilms, marine biofouling, antifouling strategies, copper nanoparticles, silver nanoparticles, metal-enzyme hybrid

## Abstract

Marine biofouling causes significant ecological, environmental, and economic impacts worldwide and has driven extensive research into effective, cost-efficient, and environmentally sustainable solutions. In this study, we develop metal nanoparticle­(NPs)-enzyme hybrids to mitigate a seven-week-old pre-formed *Cobetia marina* biofilm. These bionanohybrids consisted of copper or copper–silver NPs embedded in a protein network, and were synthesized under mild conditions using different protocols. The formation of nanostructures from 1.0 to 2.6 μm was observed in all bionanohybrids, as well as NPs with a diameter ranging from 3 to 60 nm. X-ray diffraction confirmed the presence of copper phosphate species in all hybrids, while silver phosphate or silver oxide species were identified depending on the synthesis method used. The antifouling activity of the metal bionanohybrids was evaluated against biofilms using a final bionanohybrid concentration of 250 ppm and a 6-h exposure period under dynamic conditions. Results showed a greater than 2 log decrease in culturable cell counts across all treatments. Confocal laser scanning microscopy analysis revealed significant structural disruption in the biofilms following bionanohybrid treatment, with an average reduction of 48% in biofilm thickness, 60% in total biovolume, and 71% in biovolume of viable cells. These findings confirm the significant antifouling and antibacterial activity of the bionanohybrids. Further investigation into their mechanism of action revealed that bionanohybrids induce changes in cell membranes and reduce bacterial metabolic activity.

## Graphical abstract

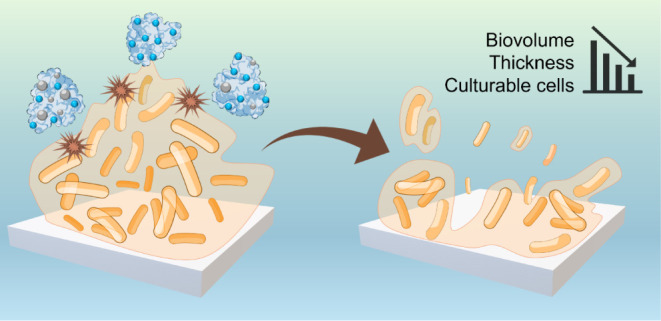

## Introduction

Marine biofouling is a global concern in aquatic environments, with several ecological, environmental, and economic impacts.^1,2^ Despite significant breakthroughs in controlling the undesirable adhesion and growth of marine fouling organisms on submerged surfaces, biofouling remains a persistent challenge.^3^ Although marine biofouling is a highly dynamic and complex process in which different agents and their interactions are involved, cell adhesion and biofilm development have been considered the initial steps in the marine fouling process.^4^ Among the diverse range of organisms that can form marine biofilms, bacteria are notably among the earliest colonizers of submerged surfaces. *Cobetia marina* has been extensively used in marine biofouling studies as a model biofilm-forming bacterium,^5–7^ due to its distinctive traits that facilitate the investigation of bacteria–surface interactions. This bacterium produces large quantities of extracellular polymeric substances (EPS)^7,8^ and exhibits gliding motility, an important feature contributing to the rapid establishment of stable and cohesive biofilms.^9,10^ Moreover, *C. marina* is a prevalent microfouler organism in marine biofilms, frequently associated with macroalgae communities and mussels.^11–13^ Owing to these characteristics, *C. marina* is commonly considered a representative marine microfouler.

Different strategies have been developed to mitigate the effects of biofilm formation. The most conventional strategies comprise physical or chemical methods that include mechanical removal, corrosion inhibitors, protective coatings, or cathodic protection.^14^ However, the most effective approaches are based on chemical methods capable of controlling biofilm development by using biocides or antifouling agents.^15^ Nevertheless, the toxicity and ecological problems associated with the use of certain chemicals limit their use. As a result, the adoption of greener alternatives has gained importance in recent years. Some examples include the use of eco-friendly inhibitors as plant derivatives,^16–18^ biosurfactants,^19^ biosynthesized metal nanoparticles (MeNPs)^20^ or MeNPs in combination with biomolecules.^21^

In this context, the use of nanomaterials has become attractive due to their significant antimicrobial efficacy and their lower environmental risk compared to other materials.^22^ In recent years, several strategies have been described in the literature for the synthesis of these MeNPs.^23,24^ One of the most promising lines focuses on the use of biological entities (enzymes) as scaffolds for the *in situ* generation of metal nanoparticles directly from metal salts, resulting in the so-called bionanohybrid materials. This green technology has advantages over other methods, where the enzyme plays an important role in the final biohybrid formed as a network that stabilizes the nanoparticles, allowing them to be in a well-homogenized distribution around the protein net, avoiding aggregation, which is a critical point in order to have an accessible reactive nanoparticle surface.^25–29^ These bionanohybrids have been successfully applied in different types of reactions,^27,29^ and particularly in the inhibition of different types of bacteria.^21,30,31^ The use of Cu and bimetallic systems against various pathogenic microorganisms—particularly bacteria associated with microbiologically influenced corrosion (MIC)—^21^ presents a promising strategy. Their application through environmentally friendly (green) synthesis methods is especially appealing for the degradation of bacterial biofilms.

In this work, different MeNPs-enzyme hybrids containing CuNPs or a combination of Cu/Ag NPs were synthesized. The enzyme (lipase from *Candida antarctica* B, CALB) used as a scaffold is a robust, cost-effective, and commercially available biocatalyst provided by Novozymes. It is employed directly, without any prior modification. This enzyme plays a key role in the *in situ* formation and stabilization of nanoparticles within the protein matrix, promoting uniform distribution and preventing aggregation. These new hybrids were characterized and applied against seven-week-old pre-formed biofilms of the Gram-negative bacterium *C. marina* (Figure 1).

**Figure 1:**
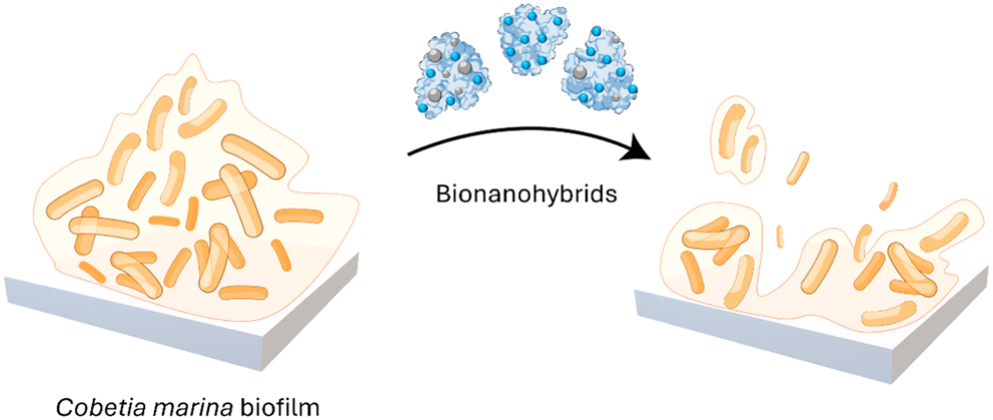
Schematic illustration of the damage caused to *Cobetia marina* biofilm by Cu and Cu/Ag bionanohybrids.

Therefore, considering that marine biofilm formation establishes the basis for the subsequent settlement of macrofouling organisms, this study aimed to develop new and sustainable materials capable of effectively preventing biofilm formation, with the goal of eventually developing coatings that can directly prevent biofilm formation on exposed surfaces.

## Materials and Methods

### Chemicals

Lipase B from *Candida antarctica* (CALB) solution was obtained from Novozymes (Copenhagen, Denmark). Copper­(II) sulfate pentahydrate [CuSO_4_·5H_2_O] was sourced from Panreac (Barcelona, Spain). Sodium dihydrogen phosphate dihydrate (NaH_2_PO_4_·2H_2_O) was purchased from Sigma-Aldrich (St. Louis, MO, USA). Silver nitrate (AgNO_3_) and disodium hydrogen phosphate dihydrate (Na_2_HPO_4_·2H_2_O) were provided by Thermo Fisher Scientific (Waltham, MA, USA).

### Synthesis of Cu Bionanohybrid

The copper hybrid was prepared as reported in our previous work.^29^ Briefly, 1.8 mL of lipase B from *Candida antarctica* (CALB) (containing 10 mg lipase/mL with >99% purity (Figure S1)) was mixed with 60 mL of sodium phosphate solution (0.1 M, pH 7). Then, 600 mg of CuSO_4_·5H_2_O was added to the mixture and the solution was stirred for 1 h at room temperature. The mixture was centrifuged for 5 min at 6440 G, and the solid obtained from the generated pellet was washed, resuspended in 20 mL of distilled water, and centrifuged again. This process was repeated twice. Finally, the resulting solid (turquoise blue) was resuspended in 2 mL of distilled water in a cryotube, frozen in liquid nitrogen, and lyophilized at −51 °C and 0.01 mbar for 16 h. Then, the metal content was determined by inductively coupled plasma-optical emission spectrometry (ICP-OES) analysis (33.33 ± 0.17 wt % Cu), after which the hybrid was named **Cu**_**33**_ **@CALB**, taking into account this amount and the protein name.

### Synthesis of Cu/Ag Bionanohybrids

A Copper–silver hybrid was synthesized starting from the previously prepared **Cu**_**33**_ **@CALB**. After washing the resulting previous solid, the blue non lyophilized solid was resuspended in 60 mL of distilled water containing 7.2 mg of AgNO_3_. The mixture was stirred at room temperature for 24 h. The final solution was then centrifuged and the new solid (grayish blue) was washed and lyophilized following the procedure described above. The ICP-OES analysis determined 31.79 ± 0.25 wt % and 0.83 ± 0.04 wt % of copper and silver contents, respectively. Thus, this hybrids was named **Cu**_**32**_ **Ag**_**1**_ **@CALB.**

A second copper–silver hybrid was synthesized by adding 300 mg of CuSO_4_·5H_2_O and 300 mg of AgNO_3_ as metal salts to 60 mL CALB solution (1.8 mL of enzyme dissolved in sodium phosphate solution (0.1 M, pH 7)). The mixture was stirred at room temperature for 24 h, and after that, the resulting solid (grayish) was recovered by centrifugation, washed, and lyophilized following the procedure described above. The amount of metal, determined by ICP-OES, was 15.46 ± 0.42 wt % Cu and 12.63 ± 0.25 wt % Ag and the hybrid was named **Cu**_**16**_ **Ag**_**13**_ **@CALB.**

### Characterization and Analytical Techniques of Bionanohybrids

Inductively coupled plasma-optical emission spectrometry (ICP-OES) was performed using an OPTIMA 2100 DV instrument (PerkinElmer, Waltham, MA, USA) to evaluate the metal content and its potential leaching. Fourier transform infrared spectroscopy (FT-IR) was performed using an FT-IR-4600 spectrophotometer (JASCO, Tokyo, Japan). X-ray diffraction (XRD) patterns were obtained using a PANalytical X’Pert Pro polycrystalline X-ray diffractometer (Malvern Panalytical Ltd., Malvern, UK) with a θ–θ setup and Cu Kα radiation and analyzed using the X’ Pert Highscore Plus program. Scanning electron microscopy (SEM) imaging was performed on a TM-1000 microscope (Hitachi, Tokyo, Japan). Transmission electron microscopy (TEM) images were obtained in an S/TEM Titan 80–300 microscope (Thermo Fisher Scientific, Waltham, MA, USA) equipped with a Cetcor Cs probe corrector and an energy dispersive X-ray spectrometer (EDS) for chemical composition analysis. The colloidal stability was studied by dynamic light scattering (DLS) using a Zetasizer detector (Malvern Panalytical Ltd., Malvern, UK). X-ray photoelectron spectroscopy (XPS) was performed using a SPECS system (SPECS Surface Nano Analysis GmbH, Berlin, Germany) under ultrahigh vacuum (pressure approximately 10^–10^ mbar) equipped with a PHOIBOS 150 9MCD analyzer and monochromatic X-ray source. Their analysis was carried out using the CasaXPS program.

### Bacterial Strain and Culture Conditions

The Gram-negative bacterium *C. marina* (DSMZ 4741) was used as a model organism for marine biofilm development.^5^ *C. marina* was previously isolated from a coastal sea sample close to Woods Hole (Falmouth, MA, USA)^32^ and purchased from the Leibniz Institute DSMZ—German Collection of Microorganisms and Cell Cultures (Braunschweig, Germany). Before the experiments, *C. marina* was streaked onto Våatanen Nine Salt Solution (VNSS) marine medium supplemented with 15 g/L agar (VWR International, Leuven, Belgium) and incubated for 18 h at 25 °C. Subsequently, individual bacterial colonies were inoculated into 100 mL VNSS medium, which is a complex salt-rich medium frequently used in marine microbiology.^5^ Following overnight incubation at 25 °C and 100 rpm (Agitorb 200ICP, Norconcessus, Ermesinde, Portugal), the bacterial culture was centrifuged (Eppendorf Centrifuge 5810R, Eppendorf, Hamburg, Germany) for 10 min at 3100 G. The pellet was resuspended in fresh VNSS medium, and the bacterial cell suspension was diluted and adjusted to a final concentration of 3.5 × 10^7^ cells/mL (OD_610 nm_ = 0.1) using a V-1200 spectrophotometer (VWR International China Co., Ltd., Shanghai, China) to perform the biofilm assays.

### Biofilm Formation Assays

Biofilm development was conducted in a long-term assay under controlled hydrodynamic conditions simulating marine environments.^33^ *C. marina* biofilms were formed on glass coupons (1 cm^2^; Vidraria Lousada, Lda, Portugal) using 12-well microtiter plates (VWR International, Carnaxide, Portugal). Glass is a common artificial submerged surface found on several devices within aquatic and marine settings, including flotation spheres, moored buoys, measuring instruments or sensors, aquaculture equipment, underwater windows of vessels, and underwater cameras.^34–36^ Glass coupons were first cleaned by immersion in a 2% (*v*/*v*) TEGO 2000 solution (Johnson Diversey, Northampton, UK) for 20 min under agitation. Subsequently, coupons were rinsed with sterile distilled water to remove any residual solution and sterilized in an autoclave at 121 °C for 15 min.^33,37^ The coupons were then fixed to the bottom of microplate wells using transparent double-sided adhesive tape and subjected to UV sterilization for 30 min. Afterward, 3 mL of bacterial suspension was added to each well, and microplates were incubated at 25 °C in an orbital shaker with a 25 mm diameter (Agitorb 200ICP, Norconcessus, Ermesinde, Portugal) at 185 rpm, achieving an average shear rate of 40 s^–1^ (similar to the shear rate value for a ship in a harbor).^38^ This setup was shown to predict the observed biofouling patterns during extended sea immersion of surfaces.^39^

Biofilm development occurred for 7 weeks (49 days), as a two-month interval for maintenance is considered the minimum duration for economically viable underwater monitoring systems.^33,35^ During this incubation time, the medium was replaced twice weekly. A total of six replicates (three biological assays with two technical replicates each) were analyzed.

### Optical Coherence Tomography for Pre-formed Biofilm Analysis

After 7 weeks of biofilm development, its structure was evaluated by Optical Coherence Tomography (OCT). Briefly, the culture medium was carefully removed and replaced with 3 mL of sterile sodium chloride solution (8.5 g/L NaCl) as a washing step to remove loosely attached cells. The microplate wells were then refilled with 3 mL NaCl solution, and 2D and 3D images from *C. marina* biofilms were captured and analyzed as previously reported,^5,33,40^ using a Thorlabs Ganymede Spectral Domain Optical Coherence Tomography system with a central wavelength of 930 nm (Thorlabs GmbH, Dachau, Germany). Since biofilms are mainly composed of water,^41^ the refractive index was set to 1.40, which is close to the refractive index of water (1.33), and representative sections from the whole coupon surface were arbitrarily chosen. For image analysis, the bottom of the biofilm was determined as the best-fitting parabola and hyperboloid, in 2D and 3D images, respectively, that connected the white pixels resulting from light reflection on the substratum surface. A Gray-value threshold that separates the biofilm from the background was considered based on the Gray-value histogram of the entire region of interest selected.^42^ The upper contour line of the biofilm was defined as the pixels with the highest distance to the bottom that had a Gray value higher than the Gray-value threshold, and which were connected to the bottom of the biofilm. Objects not connected to the bottom were excluded from the biofilm structure. For each coupon, 2D and 3D imaging was performed with a minimum of 2 fields of view to ensure the accuracy and reliability of the results obtained. The biofilm thickness, empty spaces and contour coefficient parameters were measured.

### Biofilm Treatment with Bionanohybrids

After 7 weeks of biofilm development, the *C. marina* biofilms were exposed to the different metal bionanohybrids solutions: **Cu**_**33**_ **@CALB**, **Cu**_**32**_ **Ag**_**1**_ **@CALB** and **Cu**_**16**_ **Ag**_**13**_ **@CALB**. These solutions were aseptically and freshly prepared in sterile distilled water to a final concentration of 250 ppm. The culture medium was carefully removed, and biofilms were treated with bionanohybrid solutions (3 mL per well) for 6 h under the same hydrodynamic conditions used for the biofilm formation experiments, attaining an average shear rate of 40 s^–1^. Sterile distilled water was used as the treatment control. A real marine biofilm sample was also exposed to the bionanohybrids under the same conditions (Section D. Antimicrobial effect of bionanohybrids against a real marine biofilm sample in the Supporting Information). Additionally, **Cu**_**32**_ **Ag**_**1**_ **@CALB** was incorporated into a polymeric matrix and fixed in steel coupons, and its antifouling potential against *C. marina* was evaluated under the same biofilm formation conditions over a three-week period (Section E. Antifouling effect of a bionanohybrid-functionalized surface against 3-week-old *C. marina* biofilms in the Supporting Information).

### Biofilm Analysis

After 6 h of exposure, the metal bionanohybrid solutions were removed, and the biofilms were washed with sterile NaCl solution (8.5 g/L) to eliminate any remaining residues. Subsequently, the total and culturable biofilm cells were enumerated, and the effects of the bionanohybrids on biofilm structure and bacterial viability were assessed by Confocal Laser Scanning Microscopy (CLSM). Additionally, the mechanisms of action of the different bionanohybrids were characterized by flow cytometry.

### Enumeration of Total and Culturable Biofilm Cells

To determine the number of total and culturable biofilm cells after treatment with metal bionanohybrids and sterile distilled water (control), glass coupons were detached from the microplate wells, immersed in 2 mL of sterile NaCl solution (8.5 g/L), and vortexed for 3 min to detach the adhered bacteria.^5^ Subsequently, the number of cells per cm^2^ was determined through flow cytometry by acquiring 10 μL of biofilm cell suspension at a flow rate of 10 μL/min (CytoFLEX V0–B3-R1, Beckman Coulter, Brea, CA, USA). The number of culturable biofilm cells per cm^2^ was determined by properly diluting and spreading bacterial suspensions on VNSS agar plates and incubating them overnight at 25 °C for colony-forming unit (CFU) counting.

### Confocal Laser Scanning Microscopy (CLSM)

The effect of metal bionanohybrids on *C. marina* biofilm structure and viability was evaluated by CLSM. Biofilms were stained with the Live/Dead BacLight bacterial viability kit (Invitrogen Life Technologies, Alfagene, Portugal) for 10 min in the dark.^43^ This kit contains two nucleic acid-binding stains: SYTO 9, which penetrates all cells regardless of membrane integrity (green cells), and PI, which only stains cells with compromised membranes (red cells). Biofilm samples were then inverted, mounted on a coverslip, and analyzed using a Leica DMI6000-CS inverted microscope (Leica Microsystems, Wetzlar, Germany) with a × 40 water immersion objective (Leica HCX PL APO CS; Leica Microsystems). SYTO 9 and PI signals were simultaneously acquired at different excitation and emission wavelengths. SYTO 9-stained cells were observed using a 488 nm argon laser for excitation in combination with a 500–550 nm bandpass emission filter, while PI-stained cells were detected using a 633 nm helium–neon laser in combination with a 610–680 nm bandpass emission filter. A minimum of five stacks of horizontal plane images (387.5 μm × 387.5 μm) with a *z*-step of 1 μm were acquired for each biofilm sample. Simulated 3D projections and two-dimensional (2D) sections of the biofilms were obtained using IMARIS 9.3 (Bitplane AG, Zurich, Switzerland). Biofilm biovolume (μm^3^/μm^2^) and thickness (μm) were extracted from CLSM stacks using COMSTAT2.^44^ Additionally, the Histo tool provided by ZEN lite 3.9 software (Carl Zeiss Microscopy GmbH) was used to trace the intensity values of both fluorescence signals in the z-position. Two independent experiments were conducted, each with two replicates for technical validation.

### Characterization of the Mechanisms of Action of the Metal Bionanohybrids

The mechanisms of action of the metal bionanohybrids were characterized by flow cytometry. Briefly, after treatment, biofilm cell suspensions were stained with bis­(1,3-dibutylbarbituric acid) trimethine oxonol (DiBAC_4_(3); Sigma-Aldrich, Taufkirchen, Germany) at 0.5 μg/mL to evaluate cell membrane potential, PI (Invitrogen Life Technologies, Alfagene, Lisboa, Portugal) at 1 μg/mL to assess cell membrane integrity, 5(6)-carboxyfluorescein diacetate (CFDA; Sigma-Aldrich, Taufkirchen, Germany) at 5 μg/mL to evaluate bacterial metabolic activity, and 2′,7′-dichlorofluorescein diacetate (DCFH-DA, Sigma-Aldrich, Taufkirchen, Germany) at 10 μM to detect the endogenous production of reactive oxygen species (ROS), as previous described.^5,6^ Subsequently, 20,000 cells were acquired at a flow rate of 30 μL/min in a CytoFLEX flow cytometer model V0–B3-R1 (Beckman Coulter, Brea, CA, USA). The results were analyzed using CytExpert software (version 2.4.0.28, Beckman Coulter, Brea, CA, USA) and presented as the percentage of stained cells for PI (PI­(+)-cells) and the mean intensity of fluorescence (MIF) for DiBAC_4_(3), CFDA, and DCFH-DA. At least two independent assays, each with two technical replicates, were performed.

### Statistical Analysis

Descriptive statistics were used to calculate the mean and standard deviation (SD) for the number of total and culturable biofilm cells, biofilm thickness, biovolume, and the fluorescence intensity of cells analyzed by flow cytometry. Differences between the number of total and culturable cells in the seven-week-old biofilms treated with the different metal bionanohybrids and sterile distilled water (control), and between biofilms treated with the different metal bionanohybrids were evaluated using a one-way ANOVA with Tukey’s multiple comparisons test (GraphPad Prism for Windows, version 6.01; GraphPad Software, Inc., San Diego, CA, USA). Quantitative parameters obtained from confocal microscopy analysis (biofilm thickness and biovolume) were compared using a one-way ANOVA. Differences in cell fluorescence intensity and the percentage of stained cells between each treatment condition and the control, and between each treatment condition, were evaluated using a *t*-test.

Statistically significant differences between the treatment conditions were considered for *p* values < 0.05 and represented by different lowercase letters.

## Results and Discussion

### Characterization of Enzyme–Metal Bionanohybrids

Three different metal bionanohybrids were designed and synthesized, one containing copper (**Cu**_**33**_ **@CALB)** and two different bimetallic Cu/Ag hybrids (**Cu**_**32**_ **Ag**_**1**_ **@CALB** and **Cu**_**16**_ **Ag**_**13**_ **@CALB**) (Figures 2 and 3).

**Figure 2:**
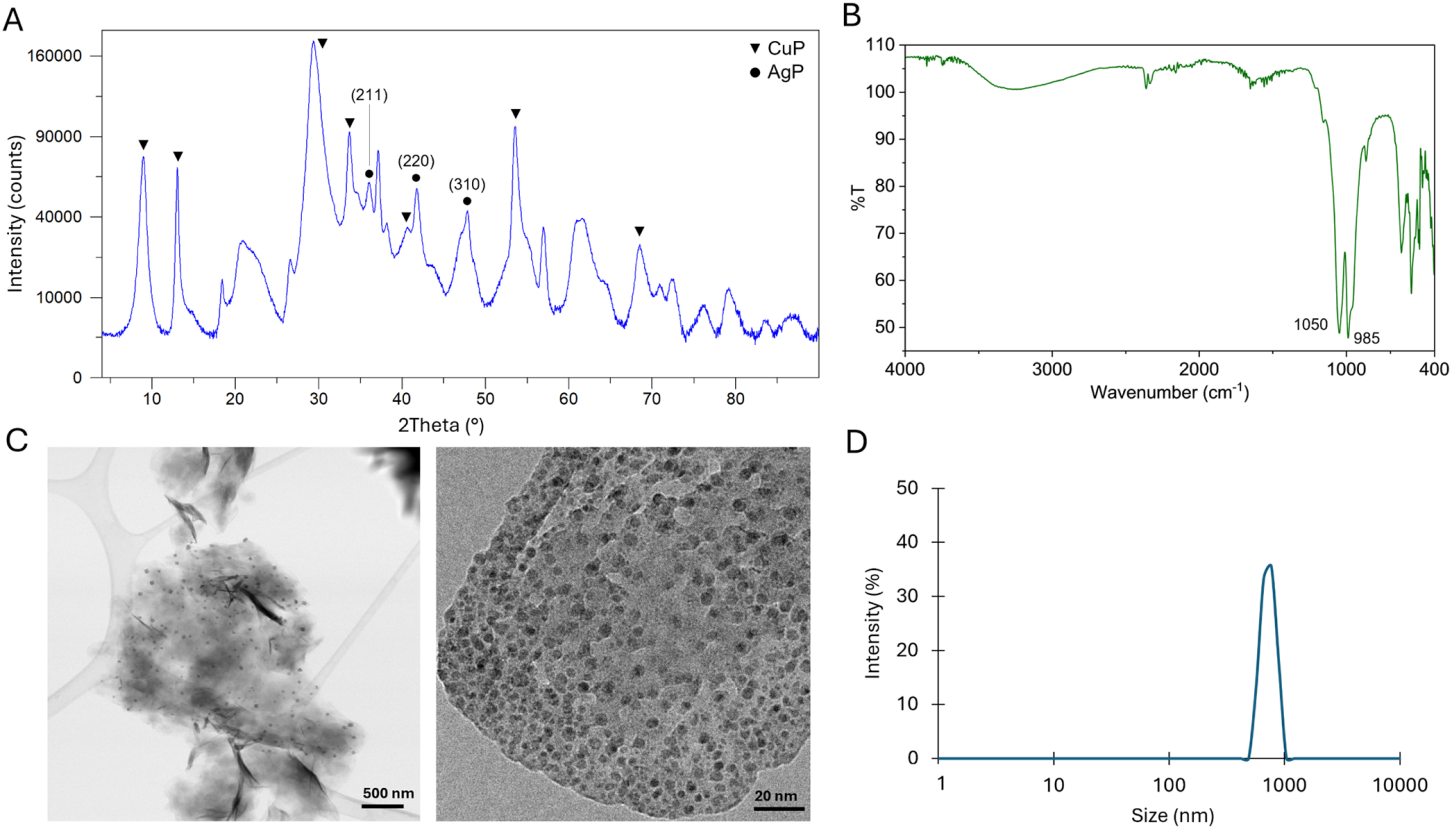
Characterization of **Cu**_**32**_ **Ag**_**1**_ **@CALB**. (A) XRD pattern. (B) FT-IR spectrum. (C) TEM images. (D) DLS measurement.

**Figure 3:**
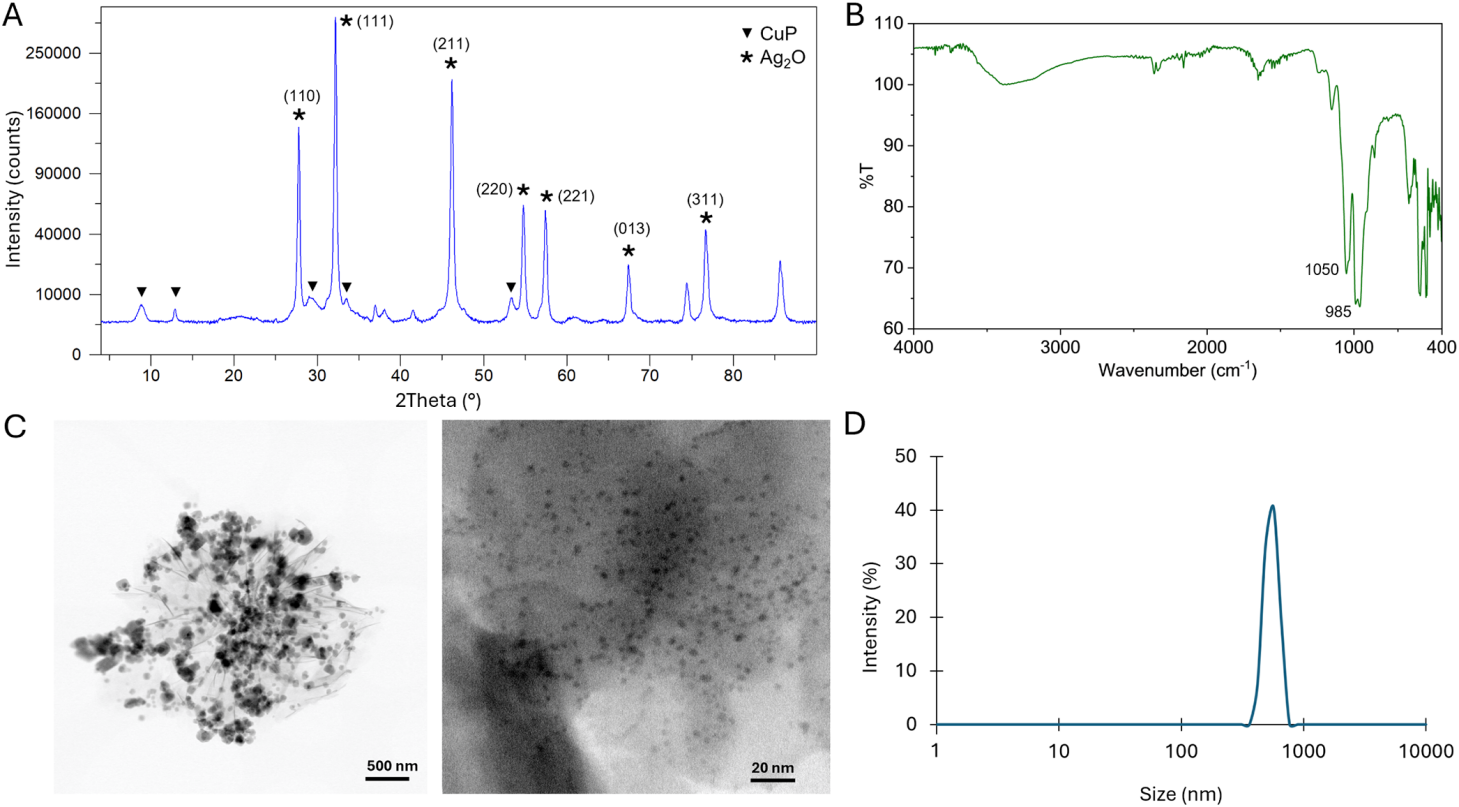
Characterization of **Cu**_**16**_ **Ag**_**13**_ **@CALB**. (A) XRD pattern. (B) FT-IR spectrum. (C) TEM images. (D) DLS measurement. CuP: copper phosphate, AgP: silver phosphate.

**Cu**_**33**_ **@CALB** was characterized in our previous studies^45,46^ and was found to be composed of microflowers of 1.0 ± 0.1 μm, with well-dispersed crystalline copper NPs with an average diameter of 4 nm, made of copper phosphate (Cu_3_(PO_4_)_2_). This distinctive microflower structure was preserved in **Cu**_**32**_ **Ag**_**1**_ **@CALB** and **Cu**_**16**_ **Ag**_**13**_ **@CALB**, as observed in SEM and TEM images (Figures 2C, 3C, S2 and S3). However, the average microflower diameters in **Cu**_**32**_ **Ag**_**1**_ **@CALB** (2.1 ± 0.7 μm) and **Cu**_**16**_ **Ag**_**13**_ **@CALB** (2.6 ± 0.4 μm) were notably larger than those observed in **Cu**_**33**_ **@CALB**, indicating an increase in microflower size with increasing silver content.^47^

XRD analyses demonstrated the presence of copper phosphate species in **Cu**_**32**_ **Ag**_**1**_ **@CALB** and **Cu**_**16**_ **Ag**_**13**_ **@CALB** (Figures 2A and 3A), that matched well with the JCPDS 00–022–0548, as was observed in **Cu**_**33**_ **@CALB**. Also, silver species were observed in the XRD patterns of the bimetallic hybrids. In the case of **Cu**_**32**_ **Ag**_**1**_ **@CALB**, the peaks (211), (220) and (310) matched well with the Ag_3_PO_4_ standard (JCPDS card no. 06–0505). However, in **Cu**_**16**_ **Ag**_**13**_ **@CALB** the silver species best match the peaks of Ag_2_O standard (JCPDS 76–1393).^48,49^

FT-IR analyses of **Cu**_**32**_ **Ag**_**1**_ **@CALB** and **Cu**_**16**_ **Ag**_**13**_ **@CALB** were performed in the range of 400 to 4000 cm^–1^ (Figures 2B and 3B). The presence of phosphate was corroborated by the observation of vibrational bands at 1050 and 985 cm^–1^, in the P–O stretching region, which aligned with those previously identified in **Cu**_**33**_ **@CALB**.

The nanostructure of the bimetallic hybrids was examined using TEM, which revealed the presence of small NPs with average diameters of 3.4 ± 0.1 and 3.2 ± 0.1 nm in **Cu**_**32**_ **Ag**_**1**_ **@CALB** (Figures 2C and S2) and **Cu**_**16**_ **Ag**_**13**_ **@CALB** (Figures 3C and S3), respectively. Additionally, larger NPs were observed in both silver–copper hybrids, particularly in **Cu**_**16**_ **Ag**_**13**_ **@CALB**, with sizes of approximately 10 nm in **Cu**_**32**_ **Ag**_**1**_ **@CALB** and ranging from 50 to 60 nm in **Cu**_**16**_ **Ag**_**13**_ **@CALB.**

It was also appreciated that the distinctive flower-like morphology of **Cu**_**33**_ **@CALB** was better preserved in **Cu**_**16**_ **Ag**_**13**_ **@CALB** than in **Cu**_**32**_ **Ag**_**1**_ **@CALB**. The average diameter of the nanostructures in **Cu**_**32**_ **Ag**_**1**_ **@CALB** (2.1 ± 0.7 μm) and **Cu**_**16**_ **Ag**_**13**_ **@CALB** (2.6 ± 0.4 μm) were notably larger than that observed in **Cu**_**33**_ **@CALB**, indicating an increase in the nanostructure size with increasing silver content.

To further characterize the structural properties, the hydrodynamic size of the hybrids was assessed using dynamic light scattering (DLS) measurements after ultrasonic dispersion in distilled water. The results revealed that **Cu**_**32**_ **Ag**_**1**_ **@CALB** exhibited a slightly higher hydrodynamic size (727.46 nm) compared to **Cu**_**16**_ **Ag**_**13**_ **@CALB** (548.06 nm) (Figures 2D and 3D). In contrast, **Cu**_**33**_ **@CALB** showed a higher degree of aggregation under the same conditions (data not shown), indicating that silver incorporation may improve dispersion stability in aqueous environments.

Then, the NPs were identified using HAADF-STEM. In both hybrids, the small NPs were attributed to copper phosphate (Figure S4A,B). Conversely, the larger NPs (>10 nm), were identified as silver (Figure S5A,B).

The surface chemistry of the bionanohybrids was investigated using X-ray photoelectron spectroscopy (XPS) (Figures S6 and S7). The full survey spectrum (Figures S6A and S7A) confirmed the existence of Cu, Ag, O elements in **Cu**_**32**_ **Ag**_**1**_ **@CALB** and Cu, Ag, O and P elements in **Cu**_**16**_ **Ag**_**13**_ **@CALB**. The high-resolution XPS spectra of Cu 2p (Figures S6B and S7B), showed two primary peaks: one at a higher energy (around 935 eV) corresponding to Cu^2+^ 2p3/2 and another at a lower energy (around 955 eV) corresponding to Cu^2+^ 2p1/2. Two additional strong satellite peaks other than the main peaks of Cu ^2+^ 2p appeared at 962.5 and 942 eV as reported by many authors.^50,51^

However, in the case of XPS spectra of Ag 3d slight differences were found in the peak of Ag 3d_5/2_, at 367 eV in **Cu**_**32**_ **Ag**_**1**_ **@CALB** (Figure S6C) whereas a peak at 367.5 eV was observed in spectra from **Cu**_**16**_ **Ag**_**13**_ **@CALB** (Figure S7C). In both cases peak correspond to Ag 3d_3/2_ was 373.5 eV. In both catalysts, these peaks are attributed to Ag+ ions, to Ag_3_PO_4_ in **Cu**_**32**_ **Ag**_**1**_ **@CALB** and Ag_2_O^52^ in **Cu**_**16**_ **Ag**_**13**_ **@CALB.**

The O 1s spectrum of **Cu**_**32**_ **Ag**_**1**_ **@CALB** (Figure S6D) showed a peak at lower binding energy peak (530.4 eV), which arises from oxygen atoms that are part of the Ag_3_PO_4_ crystal lattice, bonded to both silver and phosphorus. In **Cu**_**16**_ **Ag**_**13**_ **@CALB**, the O 1s peak appeared around 532 eV (Figure S7D), indicating a single oxygen environment in the Ag_2_O.^52^

Additionally, the enzyme-like activities of the bionanohybrids were evaluated, including oxidase-like, catalase-like, and peroxidase-like functions. The results indicated that oxidase-like activity was predominant, with **Cu**_**16**_ **Ag**_**13**_ **@CALB** exhibiting the highest activity (55.6 μmol·min^–1^ ·mg^–1^). **Cu**_**32**_ **Ag**_**1**_ **@CALB** and **Cu**_**33**_ **@CALB** showed comparable but slightly lower oxidase-like activities. In contrast, catalase-like and peroxidase-like activities were negligible across all samples (Figure S8). Kinetic parameters for oxidase activity were performed (Figure S8). K_m_ values of the different hybrids showed that the **Cu**_**32**_ **Ag**_**1**_ **@CALB** and **Cu**_**33**_ **@CALB** hybrids presented similar affinity for the substrate whereas **Cu**_**16**_ **Ag**_**13**_ **@CALB** showed higher K_m_ values, which is relative to its lower affinity for the substrate, though it is able to perform the reaction faster.

### *C. marina* Biofilm Development

*C. marina* biofilms were formed on glass under controlled hydrodynamic conditions that simulate marine environments.^33^ After 7 weeks and prior to the metal bionanohybrid treatment, the biofilm structure was evaluated using OCT (Figure 4). An average biofilm thickness of 135 μm was achieved, and the presence of three-dimensional streamers on the biofilm surface was observed. Additionally, the biofilm contour coefficient (Section C. Analysis of *C. marina* biofilms, Figure S9) – a parameter that represents the portion of the biofilm exposed to the surrounding medium, in which values close to 1 reflect a homogeneous and flat biofilm, and values higher than 1 indicate a heterogeneous structure^53^– presented an average value of 6, indicating a heterogeneous biofilm structure. Heterogeneous structures increase the surface area of the biofilm in contact with the surrounding environment, facilitating the access of nutrients and oxygen into the biofilm, thereby promoting its development.^33,40,53,54^ The OCT analysis also revealed the presence of empty spaces (Figure 4A, highlighted in blue and Section C. Analysis of *C. marina* biofilms, Figure S10), which account for 5% of the biofilm composition, with a mean size of 2620 ± 887 μm^2^. Previous studies on different marine cyanobacteria, which are key microfoulers in the marine biofouling process, growing on various surface materials (e.g., perspex, polystyrene, epoxy-coated glass, silicone hydrogel coating, and graphene-modified surfaces) have reported similar values for the percentage of empty spaces in biofilms after 7 weeks.^40,53,55^

**Figure 4:**
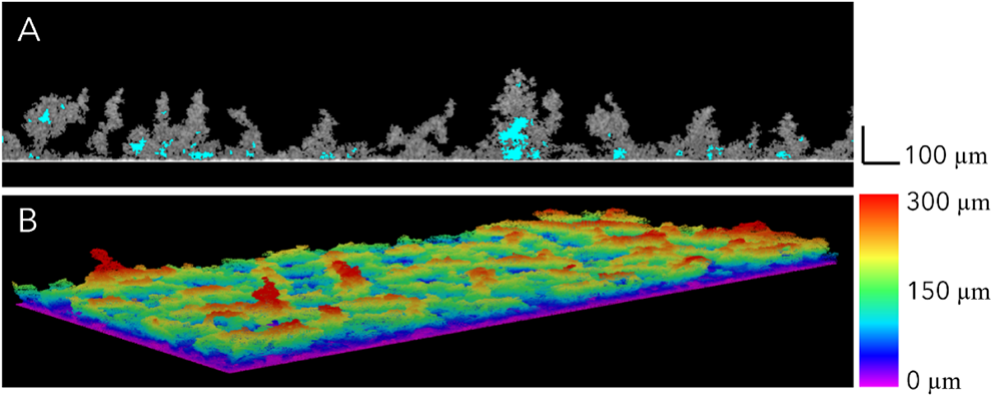
Representative 2D (A) and 3D (B) OCT images of *C. marina* biofilms formed on glass after 7 weeks and before the bionanohybrid treatments. The empty spaces of biofilms are highlighted in blue (A). The black (A) and color (B) scales show the biofilm thickness (μm).

Additionally, biofilms formed by filamentous cyanobacterial strains exhibited similar mean sizes of empty spaces,^53^ while lower mean values (ranging from 20 to 120 μm^2^) have been reported for biofilms formed by coccoid cyanobacteria.^40^ The analysis of biofilm empty spaces may provide valuable insights into the potential interaction between metal bionanohybrids and the biofilm structure. Empty spaces within biofilms influence the transport of nutrients, waste products, and antimicrobial agents. Understanding this space distribution may explain how metal bionanohybrids diffuse through the biofilm and interact with microbial cells.

### Effects of Bionanohybrid Treatments on *C. marina* Biofilms

After 7 weeks of biofilm development, the effects of the three metal bionanohybrid solutions (**Cu**_**33**_ **@CALB, Cu**_**32**_ **Ag**_**1**_ **@CALB**, and **Cu**_**16**_ **Ag**_**13**_ **@CALB**) on pre-formed *C. marina* biofilms were evaluated following their application for a 6-h period under controlled hydrodynamic conditions, using a final bionanohybrids concentration of 250 ppm. The biofilms were then analyzed for total and culturable cell counts, biofilm thickness, biovolume, and viability. Additionally, the mechanisms of action of the bionanohybrids were characterized.

The number of total and culturable biofilm cells is shown in Figure 5. The highest number of total biofilm cells was observed under the control condition (4.75 × 10^8^ ± 1.94 × 10^8^ cells/cm^2^; Figure 5A). A slight decrease in total cell counts was observed after biofilm treatment with **Cu**_**16**_ **Ag**_**13**_ **@CALB** and **Cu**_**33**_ **@CALB** (4.46 × 10^8^ ± 7.97 × 10^7^ cells/cm^2^ and 4.21 × 10^8^ ± 7.40 × 10^7^ cells/cm^2^, respectively), while treatment with **Cu**_**32**_ **Ag**_**1**_ **@CALB** resulted in a more pronounced decrease in total cell counts (3.76 × 10^8^ ± 5.62 × 10^7^ cells/cm^2^). However, no significant differences were observed in the total number of biofilm cells between the control and the treatments with the three bionanohybrids, nor between the bionanohybrid treatments themselves.

**Figure 5:**
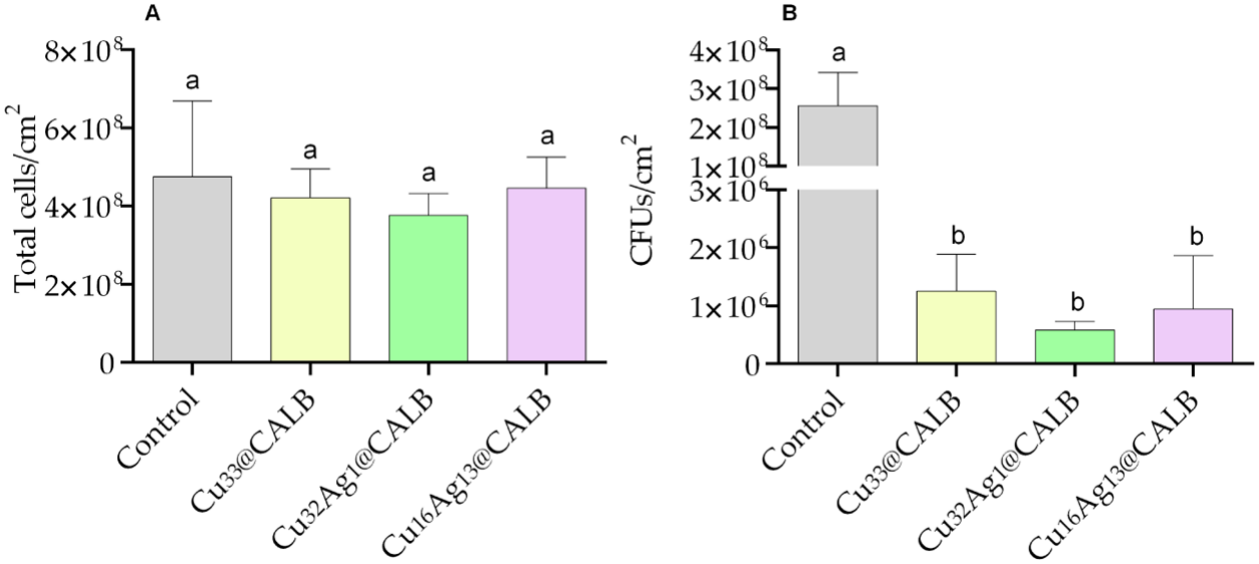
Total (A) and culturable (B) cells of seven-week-old biofilms treated with sterile distilled water (control) and the different metal bionanohybrids (**Cu**_**33**_ **@CALB, Cu**_**32**_ **Ag**_**1**_ **@CALB**, and **Cu**_**16**_ **Ag**_**13**_ **@CALB**) for 6 h under controlled hydrodynamic conditions. Mean values and SD from three biological assays with two technical replicates each are represented. Significant differences between the treatment conditions were represented by different lowercase letters (*p* < 0.05; one-way ANOVA with Tukey’s multiple comparisons test).

In contrast to the total cell counts, a significant reduction in the number of culturable cells was observed following treatments with the bionanohybrids compared to the control (Figure 5B). **Cu**_**32**_ **Ag**_**1**_ **@CALB** treatment decreased biofilm cell culturability by ∼ 2.7 log (*p* < 0.0001), whereas **Cu**_**16**_ **Ag**_**13**_ **@CALB** and **Cu**_**33**_ **@CALB** treatments decreased cell culturability by ∼ 2.4 and ∼ 2.3 log, respectively (*p* < 0.0001). Additionally, promising results were obtained regarding the application of **Cu**_**32**_ **Ag**_**1**_ **@CALB** against a real marine biofilm sample (up to 2.1 log reduction in cell culturability; Figure S11, Section D. Antimicrobial effect of bionanohybrids against a real marine biofilm sample). These findings demonstrated the antimicrobial activity of the tested metal bionanohybrid solutions. To further characterize the effect of bionanohybrid treatment on biofilm structure and viability, CLSM analysis was conducted (Figures 6 and 7).

**Figure 6:**
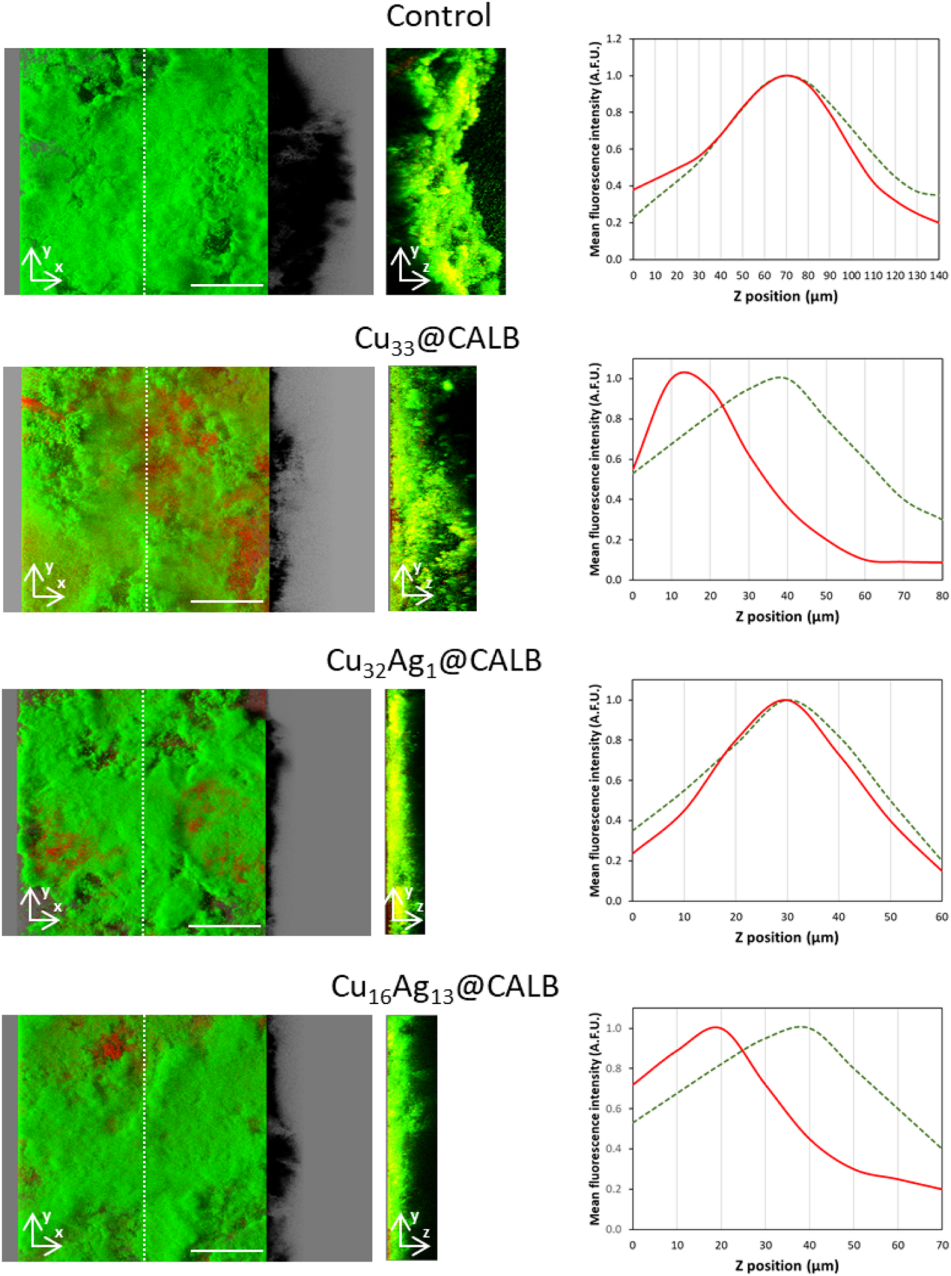
Confocal microscopy analysis of seven-week-old biofilms stained using Live/Dead BacLight bacterial viability kit, which allows the assessment of bacterial cell viability on the control and each biofilm treatment (**Cu**_**33**_ **@CALB, Cu**_**32**_ **Ag**_**1**_ **@CALB**, and **Cu**_**16**_ **Ag**_**13**_ **@CALB**). Viable and nonviable cells are shown in green and red, respectively. Representative images on the left and middle were obtained from confocal *z*-stacks using the IMARIS software; the left images present an aerial, 3D view of the biofilms (the shadow projections on the right illustrate biofilm thickness), whereas the middle images correspond to the section views in the white dotted line in the 3D images. Scale bars represent 100 μm. Histograms on the right represent the distribution of normalized green and red fluorescence intensity values (mean arbitrary fluorescence units (AFU)) along the vertical (z) biofilm position (green dotted line represents fluorescence signal, red dotted line represents fluorescence signal).

**Figure 7:**
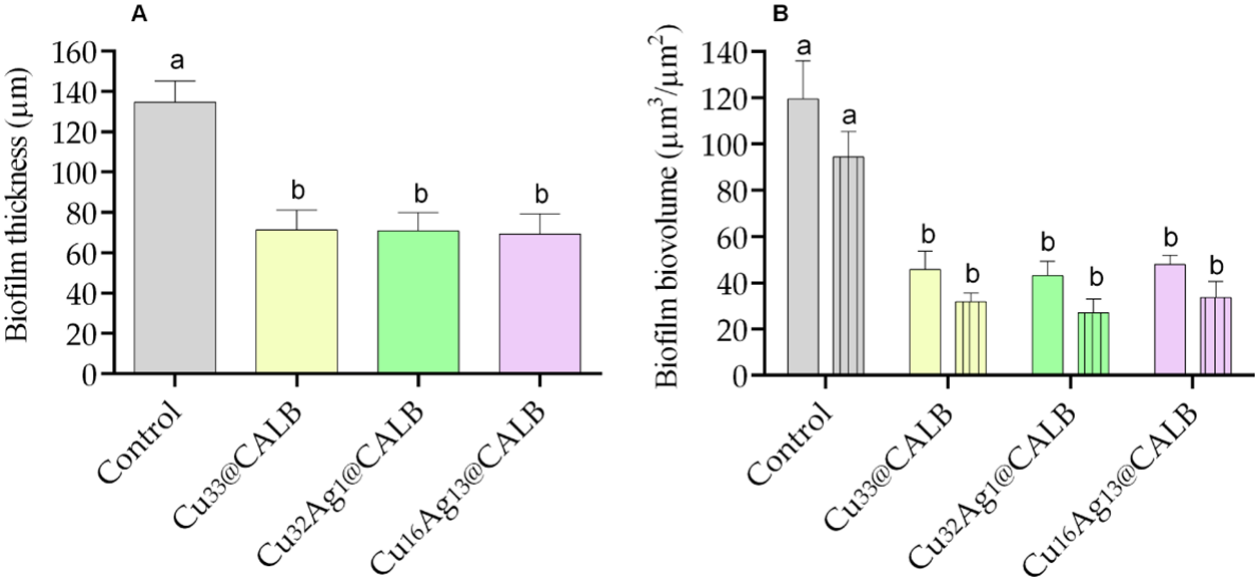
Biofilm thickness (A) and biovolume (B) of seven-week-old biofilms treated with sterile distilled water (control) and the different bionanohybrids (**Cu**_**33**_ **@CALB, Cu**_**32**_ **Ag**_**1**_ **@CALB**, and **Cu**_**16**_ **Ag**_**13**_ **@CALB)** for 6 h under controlled hydrodynamic conditions. These biofilm parameters were extracted from the CLSM stacks using COMSTAT2. Plain and patterned bars represent the total biofilm biovolume and the biovolume of viable cells, respectively. Mean values and SD from two biological assays with two technical replicates each are represented. Significant differences between the treatment conditions were represented by different lowercase letters (*p* < 0.05; one-way ANOVA with Tukey’s multiple comparisons test).

Figure 6 shows representative top- and side-view projections of the control and treated biofilms, in addition to fluorescence intensity profile graphs for each condition to show how compromised cell membranes are along the depth of the biofilm as a result of the bionanohybrids action. The control biofilms exhibited uniform and dense structures, with predominant green fluorescence, indicating a high proportion of viable bacterial cells. The fluorescence intensity profiles of the same sample showed a single, well-defined peak, suggesting the colocalization of viable and nonviable cells along the *z*-axis. In contrast, the 3D images of biofilms treated with **Cu**_**33**_ **@CALB, Cu**_**32**_ **Ag**_**1**_ **@CALB**, and **Cu**_**16**_ **Ag**_**13**_ **@CALB** revealed red areas, indicating the antimicrobial potential of these nanohybrids. Moreover, the structural integrity of the biofilm appeared compromised, with noticeable alterations in the amount of biomass and biofilm thickness. The three treated biofilms were thinner than the control, and no treatment seemed to have a greater antifouling effect. With regard to antimicrobial action, the fluorescence intensity histograms suggested differences between the bionanohybrid treatments. While the **Cu**_**32**_ **Ag**_**1**_ **@CALB** histogram presented a more dispersed profile, similar to that of the control sample, both the **Cu**_**33**_ **@CALB** and **Cu**_**16**_ **Ag**_**13**_ **@CALB** histograms showed a shift in red fluorescence intensity toward deeper layers, indicating a higher killing effect on *C. marina* cells close to the base of the biofilm. Considering the mean projected area of each of the empty spaces inside the biofilm (2620 ± 887 μm^2^), along with previous TEM and DLS data on the size of the bionanohybrids, it is expected that all compounds diffuse through the biofilm layers. However, our analysis suggests that **Cu**_**33**_ **@CALB** and **Cu**_**16**_ **Ag**_**13**_ **@CALB** have a distinct effect, affecting primarily cells located at the bottom of the biofilm. The explanation for this effect is not clear, but it may be related to different reaction kinetics of these bionanohybrids (that may be slower, thus requiring a longer residence time, which is provided by accumulation at the base of the biofilm), or a higher susceptibility of the biofilm cells located at the bottom. *C. marina* biofilms are highly heterogeneous, as demonstrated by the biofilm structural parameter contour coefficient (Section C. Analysis of *C. marina* biofilms, Figures S9 and 10). It has been shown that chemical gradients (of nutrients, oxygen, pH, signaling molecules, and waste products) exist inside them^56^ and they can induce diverse metabolic states among individual cells, promoting bacterial susceptibility, tolerance, or persistence. Moreover, we have also shown that the shear rate field on the surface of the biofilm is very heterogeneous, particularly in rough biofilms^57^ and that shear may affect the protein expression profile in these biofilms^58,59^ eventually leading to the development of a tolerant or resistant phenotype.

The quantitative results for biofilm thickness and biovolume (Figure 7) supported the qualitative CLSM analysis of the control and treated biofilms (Figure 6). Reductions in biofilm thickness by an average of 48% were observed after treatments compared to the control biofilm (*p* < 0.05; Figure 7A), and no particular bionanohybrid demonstrated superior disruptive capability over the others. Similar to thickness, reductions in both total biofilm biovolume and the biovolume of viable cells were observed after all treatments compared to the control (*p* ≤ 0.001; Figure 7B). Indeed, after bionanohybrid treatment, the total biofilm biovolume decreased by up to 60%, whereas the biovolume of viable cells was reduced by up to 71%. These results reflected the antifouling and antimicrobial activities of the tested compounds. Additionally, quantitative microscopy data (Figure 7) showed that the metal nanohybrids had similar disruptive and bacterial inactivation efficacies under the *in situ* conditions tested, as detected by cell counts (Figure 5).

The mechanisms underlying the antibacterial activity of the three tested metal bionanohybrids were characterized after biofilm treatment for 6 h under controlled hydrodynamic conditions. Biofilm cell suspensions were stained with PI, DiBAC_4_(3), CFDA, and DCFH-DA, and analyzed using flow cytometry (Figure 8).

**Figure 8:**
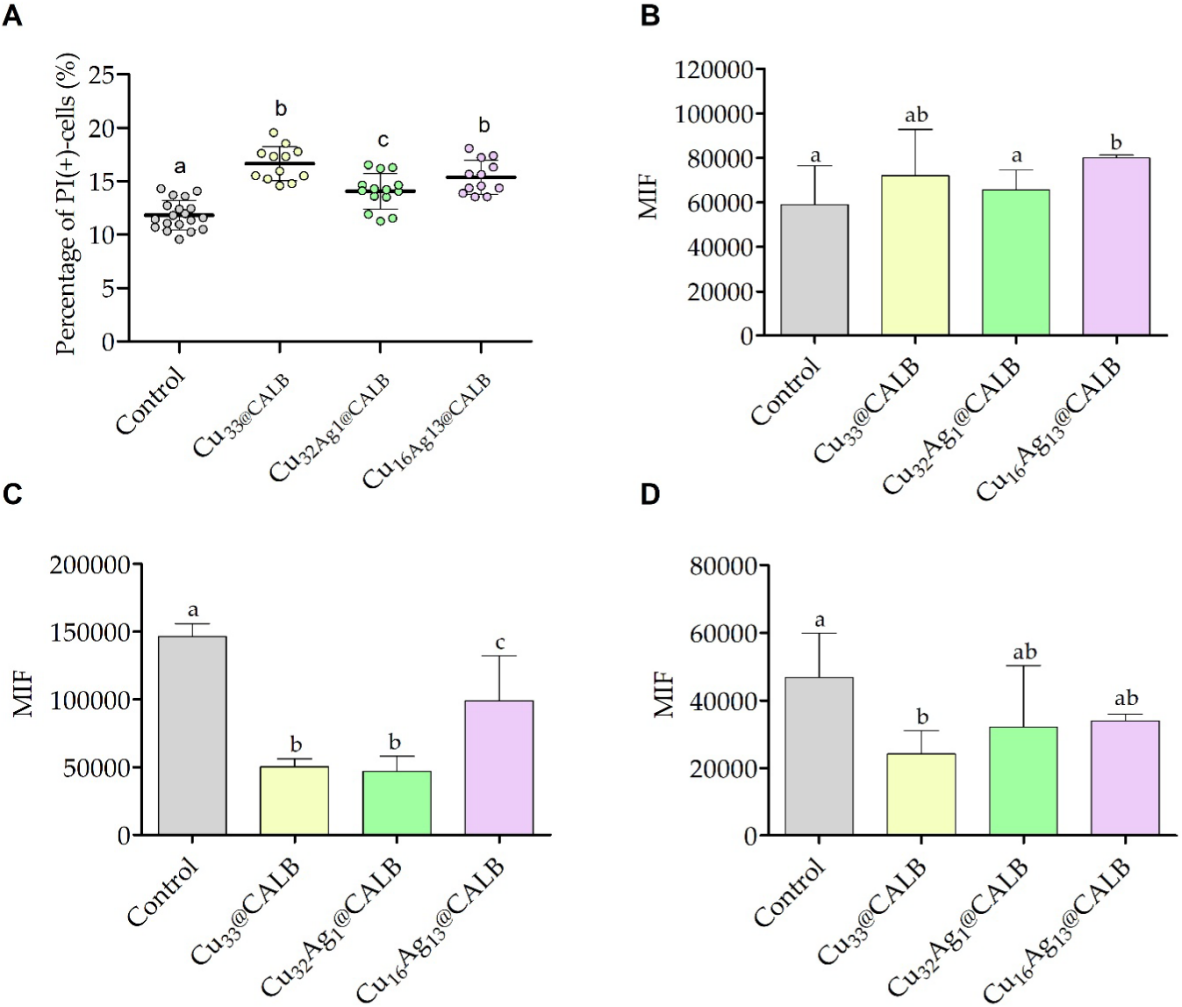
Flow cytometric analysis of *C. marina* biofilm cells treated with sterile distilled water (control) and the three different metal bionanohybrids (**Cu**_**33**_ **@CALB, Cu**_**32**_ **Ag**_**1**_ **@CALB**, and **Cu**_**16**_ **Ag**_**13**_ **@CALB)** for 6 h under controlled hydrodynamic conditions. Cells were stained with (A) PI for membrane integrity evaluation, (B) DiBAC_4_(3) for membrane potential evaluation, (C) CFDA for metabolic activity evaluation, and (D) DCFH-DA for ROS production evaluation. Flow cytometric results are presented as percentage of stained cells for PI (PI­(+)-cells) and mean intensity of fluorescence (MIF) for DiBAC_4_(3), CFDA, and DCFH-DA. Significant differences between the treatment conditions were represented by different lowercase letters (*p* < 0.05).

Flow cytometric analysis demonstrated that, under the tested conditions, **Cu**_**33**_ **@CALB**, **Cu**_**32**_ **Ag**_**1**_ **@CALB**, and **Cu**_**16**_ **Ag**_**13**_ **@CALB** metal bionanohybrids induced changes in the cell membrane, as revealed by cell staining with both PI and DiBAC_4_(3) dyes (Figure 8A and B, respectively). DiBAC_4_(3) enters depolarized cells, while PI binds to the DNA of cells with compromised membranes, both resulting in increased cellular fluorescence.^60,61^ After 6 h of treatment, biofilms exposed to **Cu**_**33**_ **@CALB**, **Cu**_**32**_ **Ag**_**1**_ **@CALB**, and **Cu**_**16**_ **Ag**_**13**_ **@CALB** showed, on average, 17%, 14% and 15% of PI-positive cells, respectively, indicating cell membrane damage (Figure 8A). Similarly, biofilm cells treated with **Cu**_**33**_ **@CALB** and **Cu**_**16**_ **Ag**_**13**_ **@CALB** and stained with DiBAC_4_(3) exhibited 1.2- and 1.4-fold higher mean intensity of fluorescence (MIF) than those exposed to distilled water (control), although this increase was statistically significant only for **Cu**_**16**_ **Ag**_**13**_ **@CALB** (Figure 8B). These data suggest that these bionanohybrids induce changes in cell membrane potential. Altogether, these findings are consistent with the reported antibacterial mechanisms of similar MeNPs-enzyme biohybrids. For instance, a tightly encapsulated invasion bactericidal mechanism was proposed for silver biohybrids, where the hybrids surrounded the Gram-negative bacteria, tightly interacted with them, and disrupted their membranes.^62^ Similarly, copper–iron bionanohybrids exhibited a comparable effect, as evidenced by the wrinkled and fragmented surfaces of dead *E. coli*, suggesting that they induced damage to the bacterial cell envelop.^63^

Additionally, cell staining with CFDA, a lipophilic substrate that is hydrolyzed to fluorescent carboxyfluorescein by esterases in the cytoplasm^64^ and is used to assess metabolic activity, demonstrated that exposure to the tested metal bionanohybrids significantly decreased the metabolic activity of biofilm cells (Figure 8C). In fact, the analysis of MIF showed that cells treated with **Cu**_**33**_ **@CALB**, **Cu**_**32**_ **Ag**_**1**_ **@CALB**, and **Cu**_**16**_ **Ag**_**13**_ **@CALB** exhibited a 2.9-, 3.1- and 1.5-fold lower MIF, respectively, than cells exposed to the control. Furthermore, the MIF of biofilm cells exposed to the bionanohybrids and stained with DCFH-DA did not increase compared to the MIF of biofilm cells exposed to the control (Figure 8D). DCFH-DA is a nonfluorescent, cell-permeable compound that is hydrolyzed by intracellular esterases to produce dichlorodihydrofluorescein (DCFH), which is then oxidized by ROS, producing a fluorescent compound.^65,66^ Therefore, these data suggest that, under the tested conditions, the bionanohybrids did not induce ROS production in *C. marina*.n

After treatment with the metal bionanohybrid solutions, the total number of biofilm cells (Figure 5A) was similar to that observed in the control. However, all bionanohybrids demonstrated antimicrobial properties against marine mature biofilms, as indicated by the reductions in the number of biofilm culturable cells (Figure 5B) and cell viability (Figure 7B) following their application. Although both biofilm parameters were reduced, the effect of the metal nanohybrids was more pronounced for culturable cell counts than for cell viability. This difference may be explained by the metabolic activity of the biofilm cells. Flow cytometric analysis revealed that *C. marina* cells significantly decreased their metabolic activity when exposed to the metal biohybrids. Therefore, although some of these cells remain viable, they may lose their culturability. In addition, treatment with the bionanohybrids resulted in a decrease in biofilm thickness (Figures 6 and 7A) and biovolume (Figures 6 and 7B). Since the total cell counts did not decrease upon exposure to the metal nanohybrids, indicating that these compounds did not remove biofilm cells, it is likely that these compounds interact with the extracellular polymeric substances (EPS) (e.g., polysaccharides), thereby reducing biofilm thickness and biovolume (Figure 7). Recently, the efficiency of zinc or silver MeNPs-enzyme hybrids has been demonstrated in the degradation of EPS in different microbial biofilms.^67^ Additionally the efficacy of copper complexes in polysaccharide oxidative depolymerization from simple disaccharides to more complex structures (cellulose, chitin), mimicking lytic polysaccharide monooxygenases, has also been reported.^68^ These findings are in line with our results for Cu and Cu/Ag bionanohybrids, underlining their potential as effective antibiofilm agents.

Furthermore, with the aim of real-world application, a representative bionanohybrid (**Cu**_**32**_ **Ag**_**1**_ **@CALB**) was incorporated into a polymeric matrix (polyurethane waterproof coating (UPC 739240462223)) applied to steel coupons, and its antifouling potential against *C. marina* was evaluated under hydrodynamic conditions simulating marine environments over a three-week period (see “Section E. Antifouling effect of a bionanohybrid-functionalized surface against 3-week-old *C. marina* biofilms” in the Supporting Information for detailed information). Bionanohybrid-functionalized surfaces reduced by 24% biofilm cell culturability and by 11% biofilm thickness compared to the control surface (Figure S12). These results highlight the antifouling potential of metal bionanohybrids, even when incorporated into polymeric surfaces, supporting their use as innovative marine antifouling coatings. This is just a proof of concept regarding the antimicrobial effect of the surface and that further optimizations in surface preparation (bionanohybrid concentration and functionalization reaction) will be made to enhance its antimicrobial activity.

Moreover, to assess the potential metal leaching from the bionanohybrid solutions and the bionanohybrid-functionalized surface, both were washed with ultrapure water, which as then analyzed by Inductively Coupled Plasma Optical Emission Spectroscopy (ICP-OES) (see Section F. Metal leaching assessment by Inductively Coupled Plasma Optical Emission Spectroscopy (ICP-OES) in Table S1. Data showed that after washing, **Cu**_**33**_ **@CALB**, **Cu**_**32**_ **Ag**_**1**_ **@CALB** and **Cu**_**16**_ **Ag**_**13**_ **@CALB** released a maximum of 0.020 ppm of Cu. In contrast, **Cu**_**32**_ **Ag**_**1**_ **@CALB** and **Cu**_**16**_ **Ag**_**13**_ **@CALB** released 0.030 and 0.200 ppm of Ag, respectively. According to the World Health Organization, the acceptable drinking water limits for cooper and silver are 2 ppm^69^ and 0.1 ppm,^70^ respectively. Therefore, the detected Cu levels are within acceptable limits, whereas the Ag levels for **Cu**_**16**_ **Ag**_**13**_ **@CALB** exceed the established thresholds. When **Cu**_**32**_ **Ag**_**1**_ **@CALB** nanohybrid was incorporated into a polymeric matrix, no Ag was detected in the washing water, demonstrating that immobilizing compounds on polymeric surfaces is a promising approach to prevent metal leaching.

## Conclusion

Different Cu and Cu/Ag bionanohybrids were synthesized using a simple and eco-friendly methodology and evaluated as antibiofouling agents against *C. marina* pre-formed biofilms. The bionanohybrids presented different metal content, metallic species, and NPs sizes according to the synthetic protocol performed. The results showed a decrease in culturable cell counts in biofilms after treatment with bionanohybrids, with a reduction of more than 2-log for the most effective one, **Cu**_**32**_ **Ag**_**1**_ **@CALB**. In addition, the hybrids reduced biofilm thickness by 48% and biovolume by 60%, indicating a strong antifouling effect. Further analysis of the mechanism of action suggested that the hybrids induce changes in cell membranes and reduce bacterial metabolic activity. Based on these results, these bionanohybrid solutions present an effective, eco-friendly alternative that can potentially control biofilms in marine environments. Indeed, the straightforward synthetic protocol, which does not require expensive equipment for production, combined with the relatively low-cost raw materials (e.g., enzyme prices around $70–80 per kg), has demonstrated the scalability of the material in certain instances. These advantages make it an ideal candidate for future research aimed at incorporating it into coatings or surfaces for marine applications.

## Supplementary Material



## Notes

Characterization analysis of bimetallic bionanohybrids (TEM and HAADF-STEM imaging and STEM-EDX analysis, XPS analyses), enzyme-like activities assays of bionanohybrids (experimental part, Michaelis–Menten plots, results of specific activities), representative 2D and 3D OCT images of *C. marina* biofilms formed on glass after 7 weeks, demonstrating the biofilm contour and the spatial distribution of pores, antimicrobial effect of bionanohybrids against a real marine biofilm sample, antifouling effect of a bionanohybrid-functionalized surface against *C. marina* biofilms, and metal leaching assessment by ICP-OES (PDF)
