# Supplementary material for: Effect of Metal Bionanohybrids on Pre-formed Marine Biofilms
Source: ACS Appl Bio Mater. 2025 Oct 3;8(10):9451–63. doi: 10.1021/acsabm.5c01575 (PMC12541695; doi:10.1021/acsabm.5c01575)
Supplement: Supplementary file 1 [file mt5c01575_si_001.pdf]

Supporting Information

Effect of Metal Bionanohybrids on Pre-formed Marine Biofilms

Clara Ortega-Nieto[a,b], Maria J. Romeu[b,c], Rita Teixeira-Santos[b,c], Luciana C. Gomes[b,c], Filipe J. Mergulhão[b,c,*], Jose M. Palomo[a,*]

[a] Instituto de Catalisis y Petroleoquimica (ICP), CSIC, c/Marie Curie 2, 2, 28049 Madrid, Spain

[b] LEPABE - Laboratory for Process Engineering, Environment, Biotechnology and Energy, Faculty of Engineering, University of Porto, Rua Dr. Roberto Frias, 4200-465, Porto, Portugal

[c] ALiCE - Associate Laboratory in Chemical Engineering, Faculty of Engineering, University of Porto, Rua Dr. Roberto Frias, 4200-465, Porto, Portugal

**Corresponding authors:*

Emails: josempalomo@icp.csic.es, filipem@fe.up.pt

A. Bionanohybrids characterization

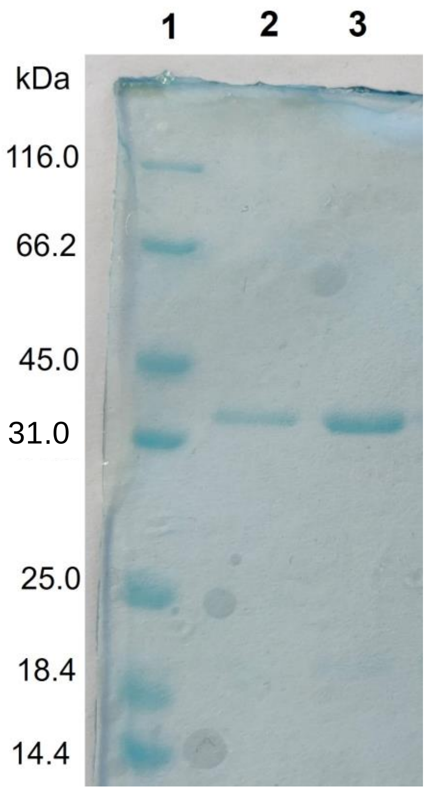

Figure S1. SDS-PAGE of commercial CALB. 1) Molecular weight marker; 2) CALB solution at 0.1 mg/mL in water; 3) CALB solution at 0.3 mg/mL in water.

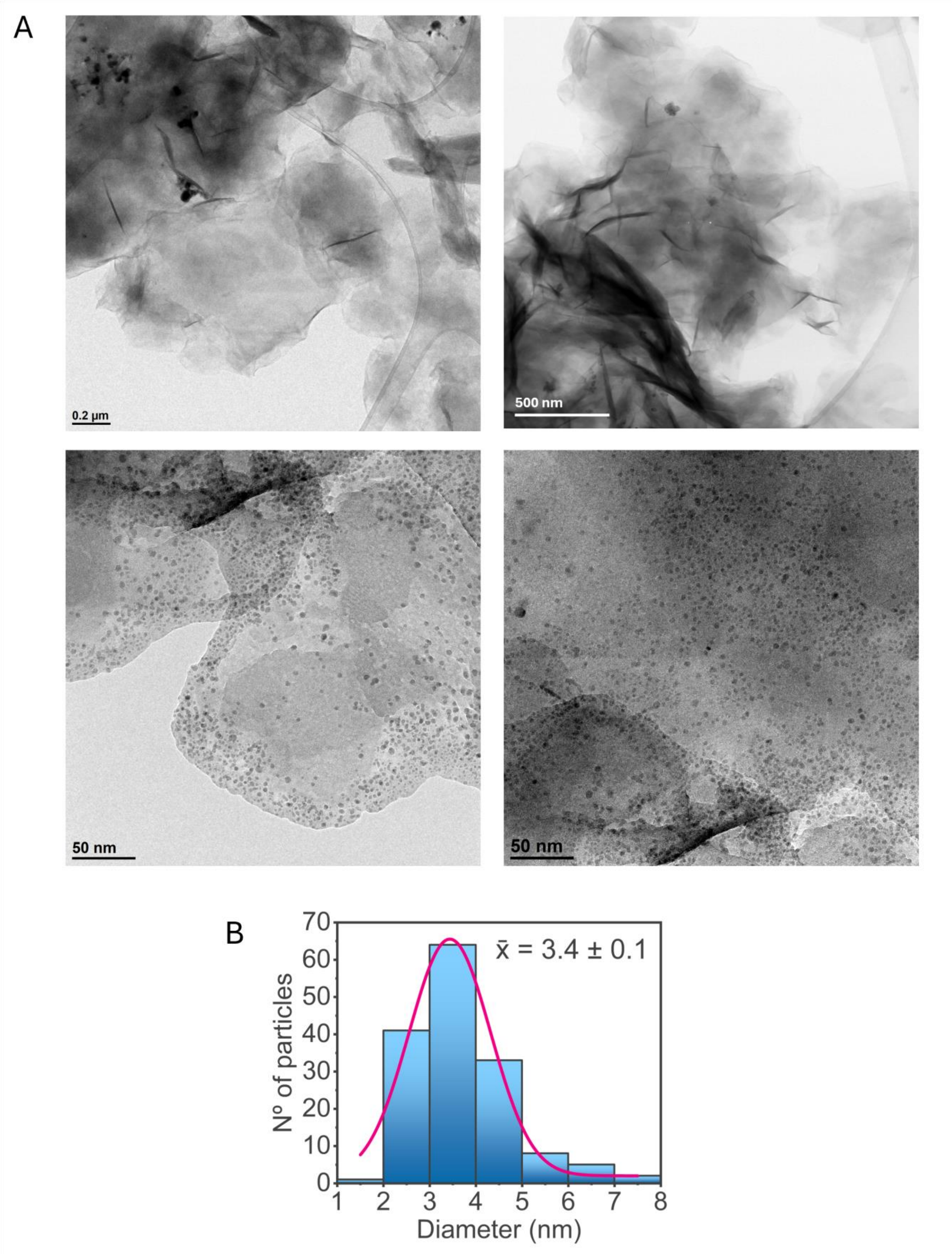

Figure S2. A) TEM images of $\mathbf{Cu_{32}Ag_1}$**@CALB**. B) Particle size distribution fitted by a Gaussian curve of $\mathbf{Cu_{32}Ag_1}$**@CALB.**

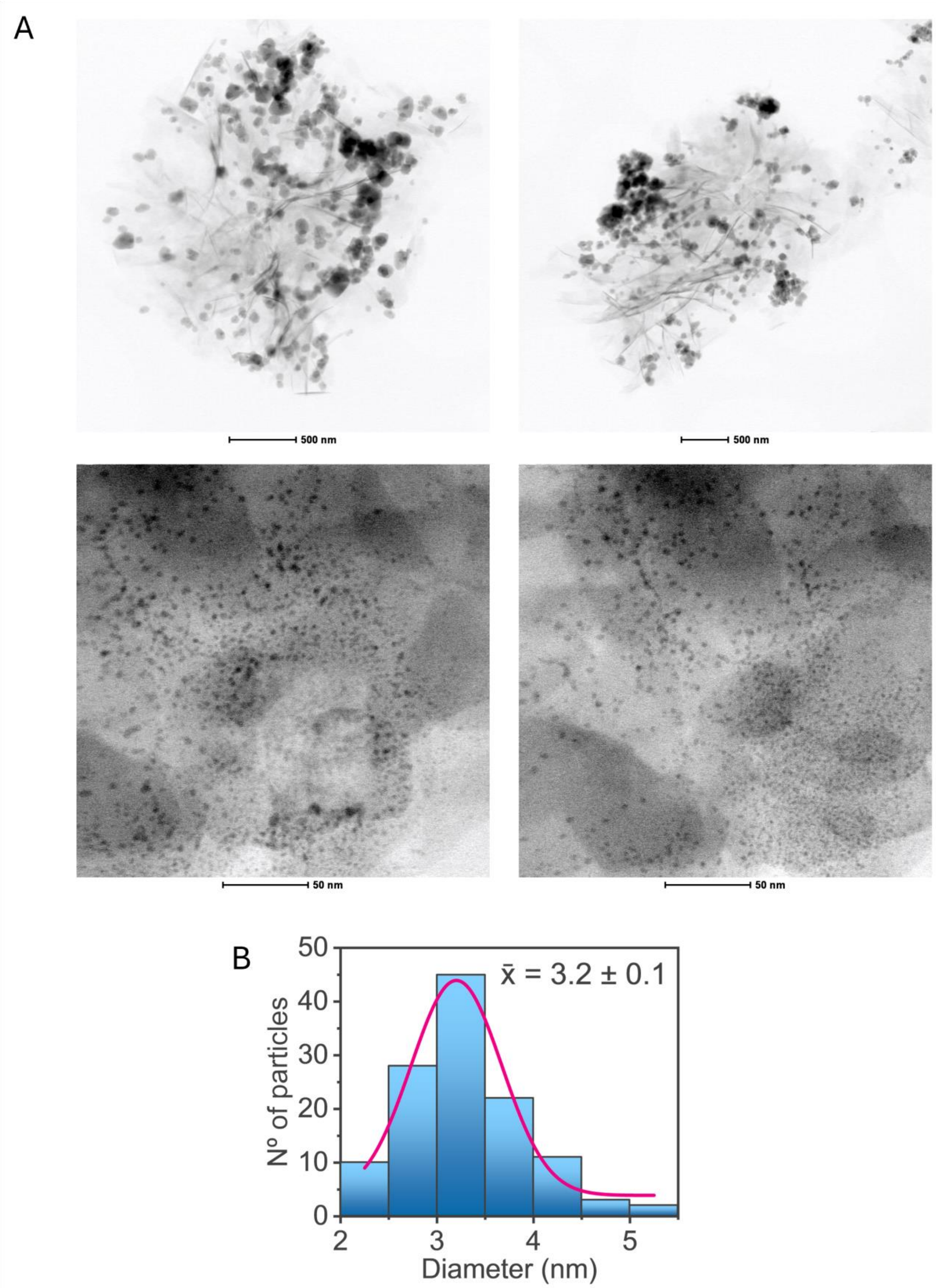

Figure S3. A) TEM images of **$Cu_{16}Ag_{13}$@CALB**. B) Particle size distribution fitted by a Gaussian curve of **$Cu_{16}Ag_{13}$@CALB**

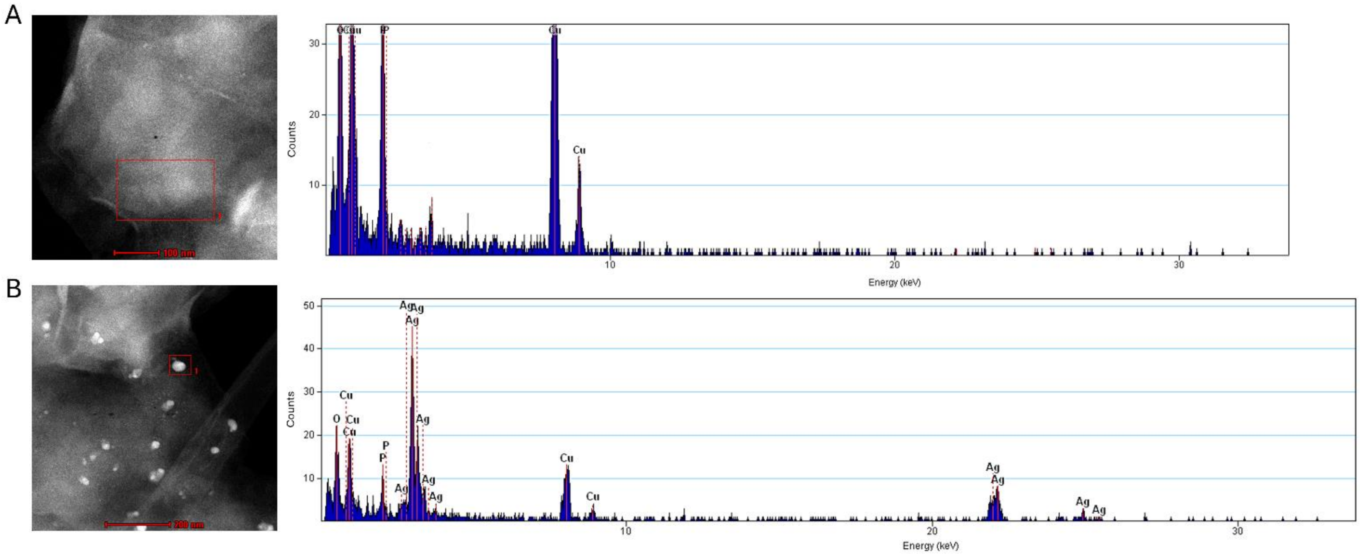

Figure S4. HAADF-STEM imaging (left) and STEM-EDX analysis (right) of **$Cu_{32}Ag_1$@CALB**. (A) Cu particles. (B) Ag particles. The nickel signal corresponds to the grid used for the experiment.

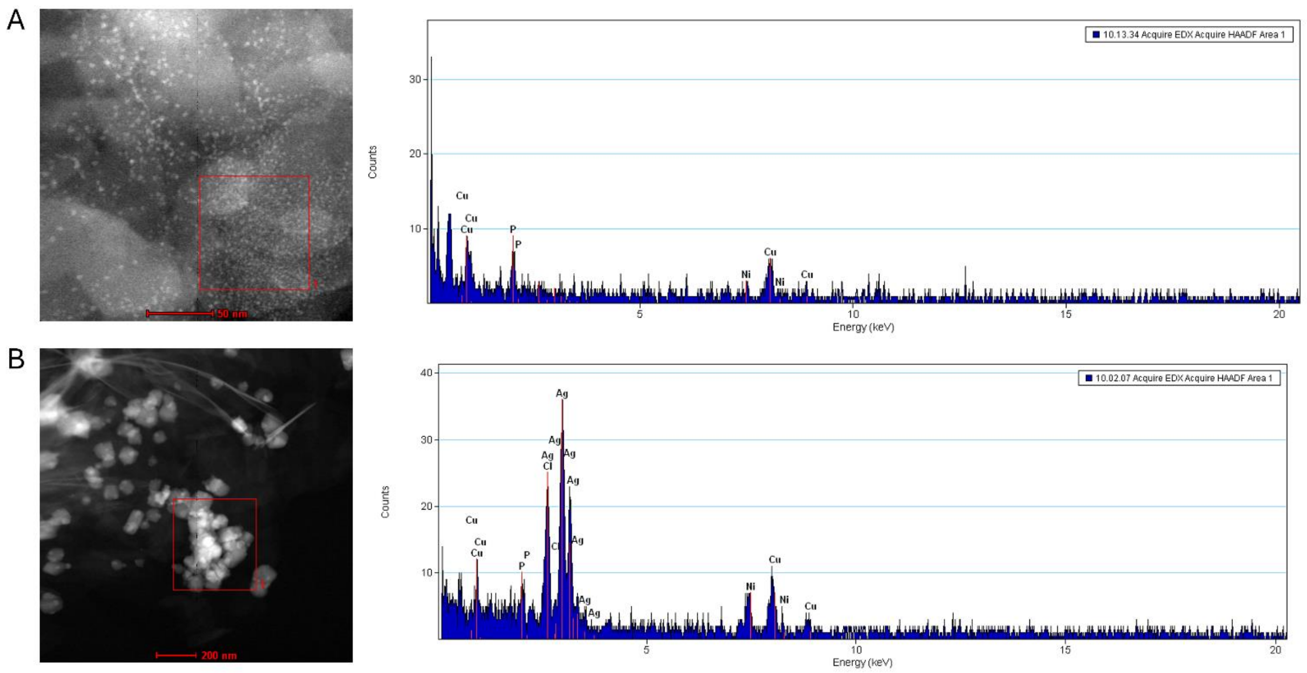

Figure S5. HAADF-STEM imaging (left) and STEM-EDX analysis (right) of **$Cu_{16}Ag_{13}$@CALB**. (A) Cu particles. (B) Ag particles. The nickel signal corresponds to the grid used for the experiment.

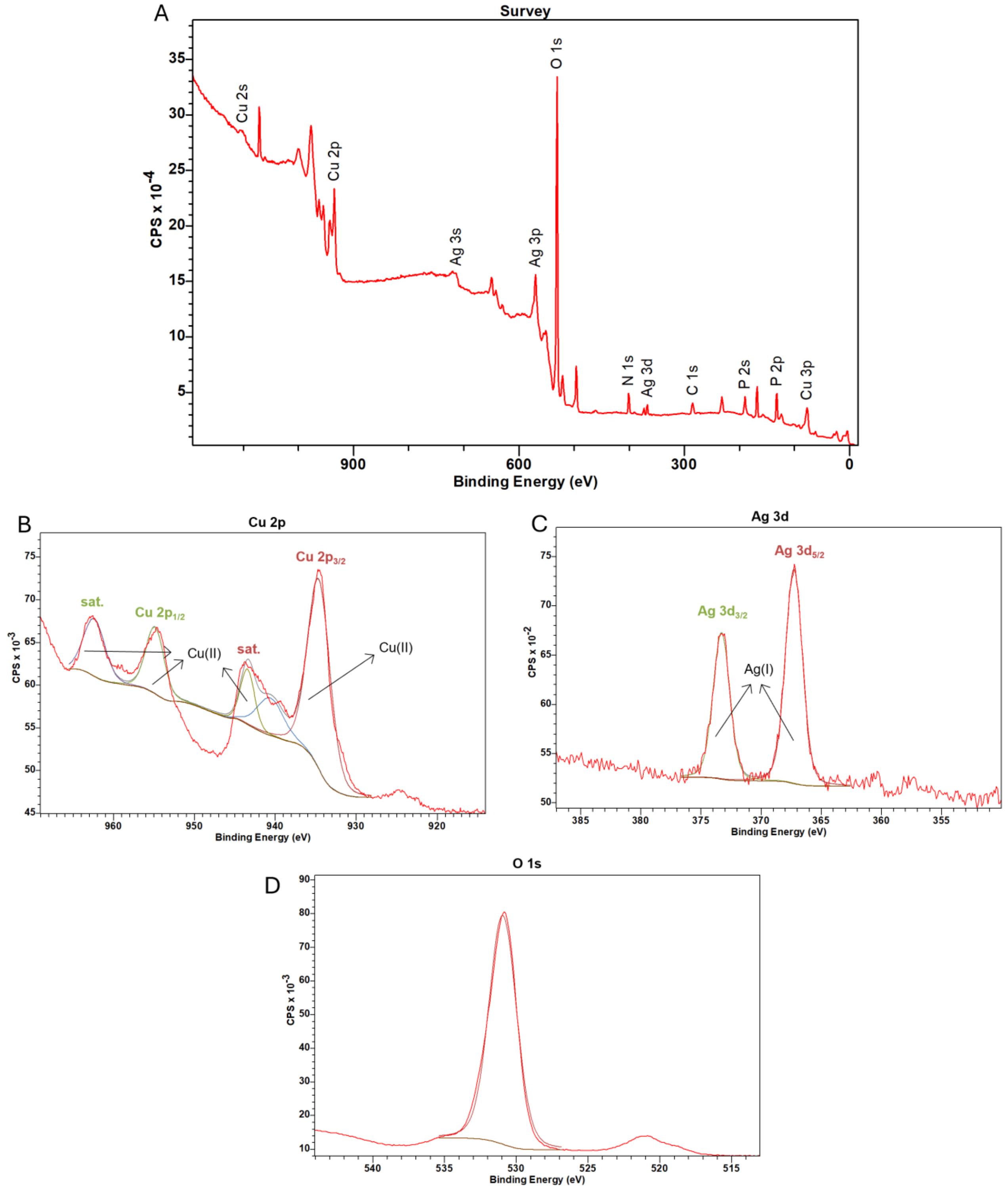

Figure S6. XPS spectrum of **$Cu_{32}Ag_1$@CALB**. A) Survey. (B) Cu 2p. (C) Ag 3d. (D) O 1s.

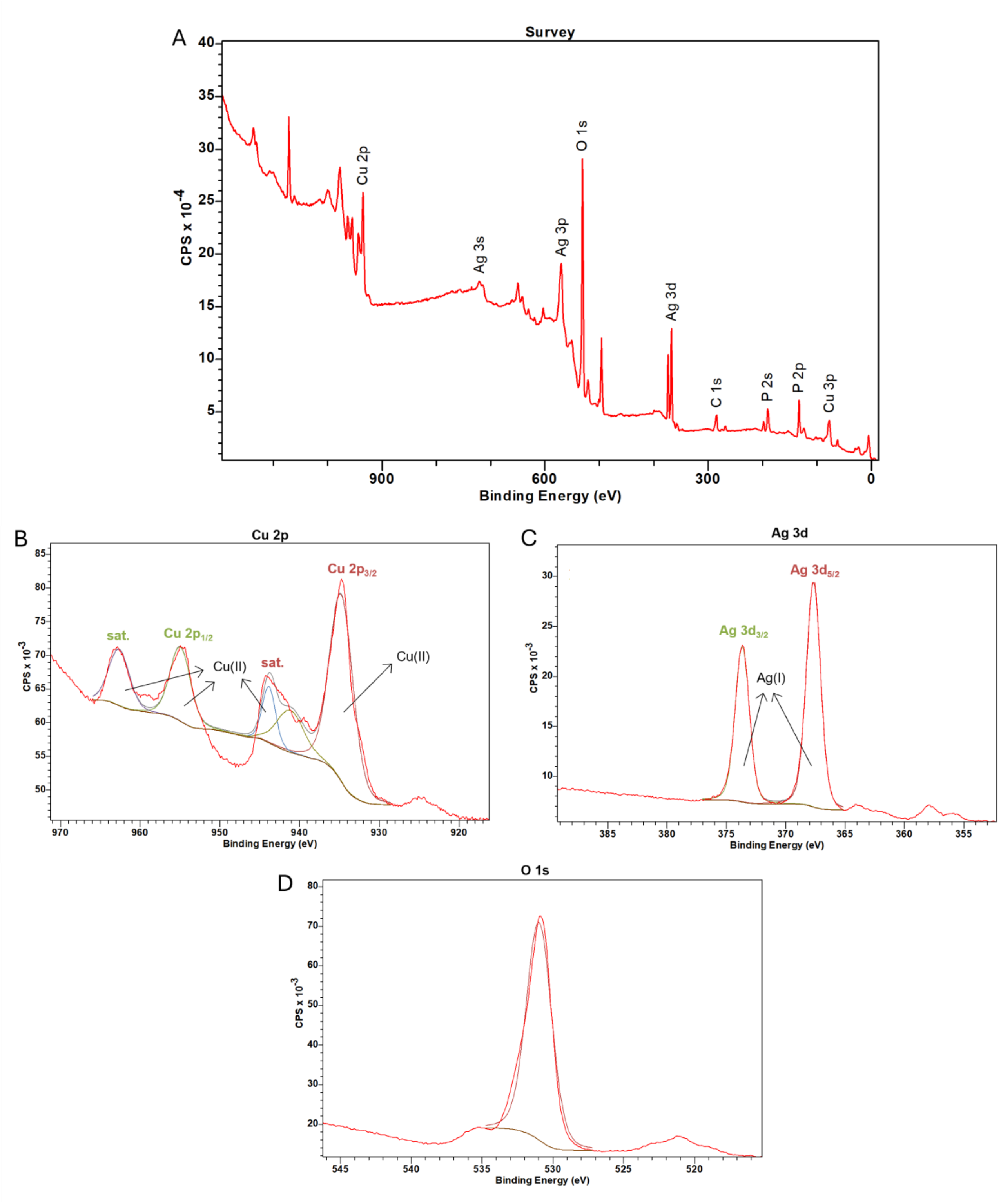

Figure S7. XPS spectrum of **$Cu_{16}Ag_{13}$@CALB**. A) Survey. (B) Cu 2p. (C) Ag 3d. (D) O 1s.

B. Bionanohybrids enzyme-like activities

Oxidase-like activity assay

10 mL of 0.5, 1 or 2 mM p-aminophenol (pAP) solution was prepared in distilled water containing H_2O_2 (0.5%, v/v). 3 mg of the bionanohybrid was used to catalyze the reaction. The mixture was stirred at room temperature under magnetic agitation. Reaction progress was monitored by HPLC analysis of samples collected at different times. Samples were centrifuged and diluted 1:10 with the mobile phase prior to injection. Experiments were performed in duplicated.

Catalase-like activity assay

10 mL of a 50 mM hydrogen peroxide (H_2O_2) solution was prepared in distilled water. To initiate the reaction, 4 mg of the bionanohybrid catalyst was added, and the mixture was stirred at room temperature under magnetic agitation. The degradation of H_2O_2 was monitored by UV-Vis spectroscopy at 240 nm using 1 cm quartz cuvettes. Aliquots were taken at various time intervals, centrifuged, and diluted 1:1 prior to measurement. Experiments were performed in duplicated.

Peroxidase-like activity

2 mL of a 0.5 mM 2,2'-Azino-bis(3-ethylbenzothiazoline-6-sulfonic acid diammonium salt (ABTS) solution was prepared in a 1:1 (v/v) ratio of 0.1 M sodium citrate buffer (pH 5) and 0.1 M sodium phosphate buffer (pH 5), containing H_2O_2 (0.5%, v/v). 2 mg of the bionanohybrid was added to initiate the reaction, and the mixture was stirred at room temperature under magnetic agitation. The reaction was followed by UV-Vis spectroscopy at 420 nm using plastic cuvettes of 1 cm. Samples were collected at different times, centrifuged and analysed.

Analytical techniques

Chromatographic analyses were performed on an HPLC system equipped with a pump (PU-4180, JASCO, Tokyo, Japan) and a UV-4075 UV-Vis detector (JASCO, Tokyo, Japan) in isocratic mode at ambient temperature. A Gemini-NX C18 column (5µm, 110Å, 250 × 4.6 mm), a mobile phase of ultrapure water and acetonitrile (90:10) at pH 4, an injection volume of 10 µL, a flow rate of 0.7 mL/min and UV detection at 210 nm were used. Spectrophotometric analyses were run on V-730 spectrophotometer (JASCO, Tokyo, Japan).

Specific activity determination

In the spectrophotometric assays, the dAbs/dt value was calculated. Then, the specific activity (U/mg) was obtained using the following equation:

$$SA(\mu mol/min \cdot mg) = \frac{dAbs}{dt} \cdot \frac{V}{\varepsilon \cdot l \cdot catalyst\ mass\ (mg)}$$

The molar extinction coefficients (ε) used were 43.6 $M^{-1}cm^{-1}$ for the catalase activity and 36,000 $M^{-1}cm^{-1}$ for peroxidase activity.

In the chromatographic assay, the dc/dt value was calculated. Then, the specific activity (U/mg) was obtained using the following equation:

$$SA(\mu mol/min \cdot mg) = \frac{dc}{dt} \cdot \frac{V}{catalyst\ mass\ (mg)}$$

a)

Hybrid	Oxidase-like SA (μmol/min·mg)	Catalase-like SA (μmol/min·mg)	Peroxidase-like SA (μmol/min·mg)
$Cu_{16}Ag_{13}$@CALB	55.62	0.0184	0.0000
$Cu_{32}Ag_{1}$@CALB	23.06	0.0043	0.0000
Cu_{33}@CALB	33.47	0.0000	0.0000

b)

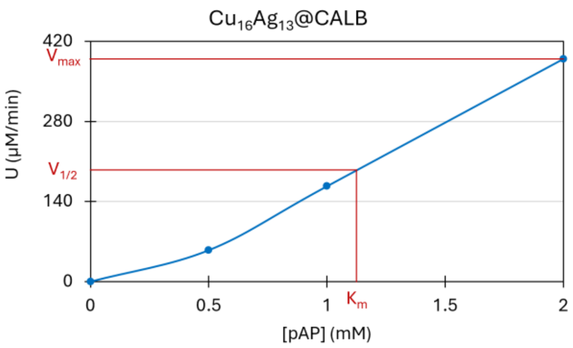

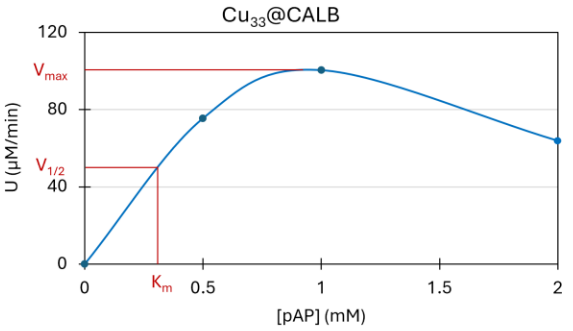

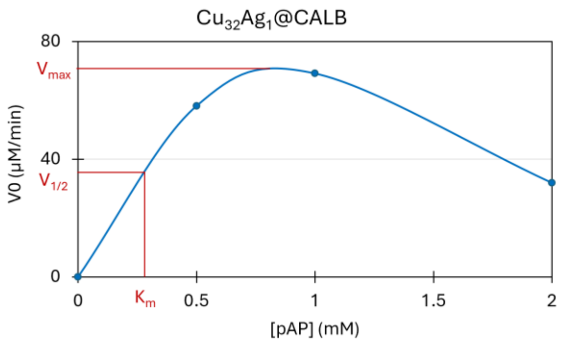

Hybrid	V_{max} (μM/min)	$V_{1/2}$ (μM/min)	K_m (mM)
$Cu_{16}Ag_{13}$@CALB	≥ 390	≥ 195	≥ 1.12
$Cu_{32}Ag_{1}$@CALB	71.0	35.5	0.28
Cu_{33}@CALB	100.0	50.0	0.31

Figure S8. a) Enzyme-like specific activities of the hybrids in aqueous media. b) Michaelis-Menten kinetics of the oxidase-like activity of the different hybrids.

C. Analysis of *C. marina* biofilms

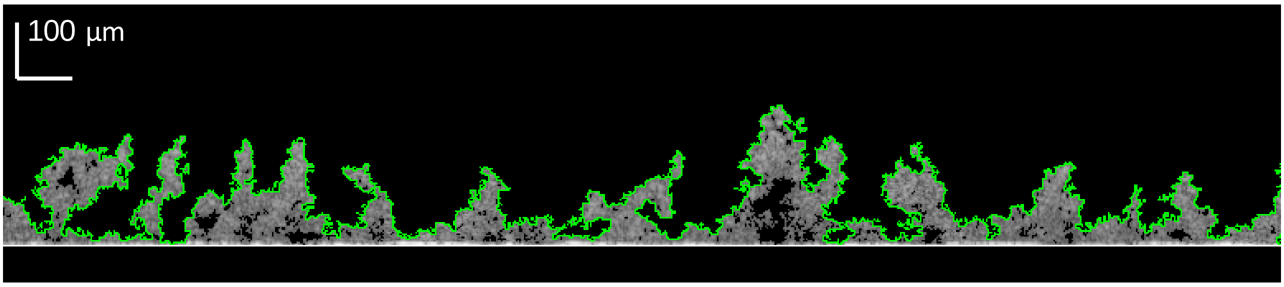

Figure S9. Representative 2D OCT image of *C. marina* biofilms formed on glass after seven weeks and before the bionanohybrid treatments. The biofilm contour (fraction of the biofilm exposed to the surrounding medium) is highlighted in green and allows the determination of the contour coefficient, which is defined as the number of voxels connected to the background (green line) divided by the number of voxels in a horizontal plane. The white scale shows the biofilm thickness (µm).

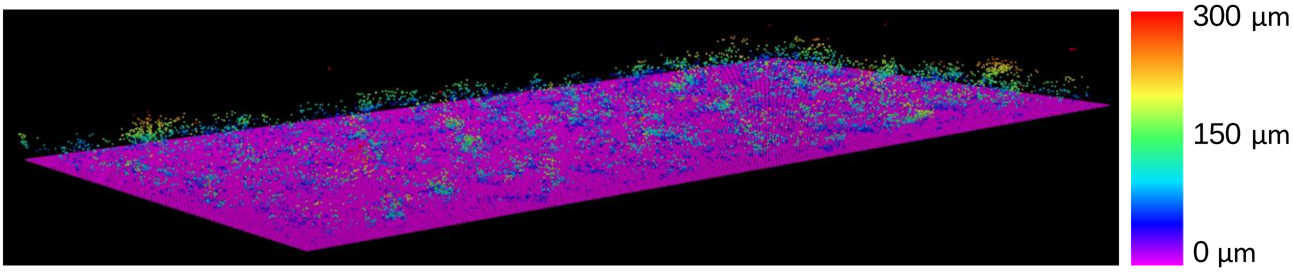

Figure S10. Representative 3D OCT image of the spatial distribution of pores in *C. marina* biofilms formed on glass after seven weeks and before the bionanohybrid treatments. The color scale shows the distance from the substrate surface.

D. Antimicrobial effect of bionanohybrids against a real marine biofilm sample

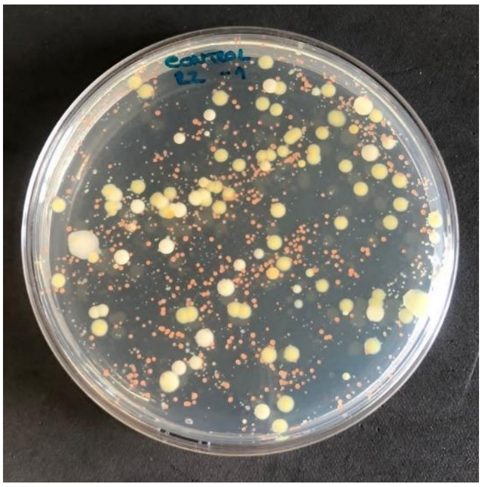

Figure S11. Microbial species diversity observed on PCA medium following the spread of the real biofilm sample collected from a pier at Freixo Marina in Porto, Portugal.

A real biofilm sample was scraped from a pier at Freixo Marina (41.137327 N 8.572064 W) in Porto, Portugal. In the laboratory, the sample was divided into four equal portions (3.65 ± 0.19 mg each). Two portions served as biofilm controls and were treated with sterile distilled water, while the other two were treated with a 250 ppm solution of the metal bionanohybrid **$Cu_{32}Ag_1$@CALB** (as a representative bionanohybrid) for 6 h under controlled hydrodynamic conditions, following the same protocol used for *in vitro* assays. After treatment, each biofilm sample was suspended in 2 mL of sterile saline solution and vortexed for 3 min. Serial dilutions were then prepared, and the microbial suspensions were spread onto Plate Count Agar (PCA) plates and incubated overnight at 25 °C. After **$Cu_{32}Ag_1$@CALB** treatment, data showed a reduction in microbial culturability of up to 2.1 log, confirming the *in vitro* antimicrobial activity of the bionanohybrid solution.

E. Antifouling effect of a bionanohybrid-functionalized surface against 3-week-old *C. marina* biofilms

Steel coupons used as control and functionalized surfaces (with polyurethane waterproof coating (UPC 739240462223) (1 cm^2) were UV-sterilized for 30 min and fixed to the wells of 12-well microtiter plates using transparent double-sided adhesive tape. *C. marina* biofilm formation on these surfaces was assessed over three weeks (21 days), at 25 °C, under hydrodynamic conditions that mimic marine environments. Biofilm thickness was evaluated by Optical Coherence Tomography (Thorlabs Ganymede Spectral Domain OCT system with a central wavelength of 930 nm, Thorlabs GmbH, Dachau, Germany) through three-dimensional biofilm imaging. To quantify the number of biofilm culturable cells, each surface was removed from the microplate wells, immersed in 2 mL of sterile saline solution (8.5 g/L) and vortexed for 3 min to detach the adhered bacteria. The number of biofilm culturable cells was assessed by spreading the bacterial suspensions on VNSS agar plates, followed by overnight incubation at 25 °C for CFU counting.

Bionanohybrid-functionalized surfaces reduced by 24% biofilm cell culturability and by 11% biofilm thickness compared to the control surface (Figure S12).

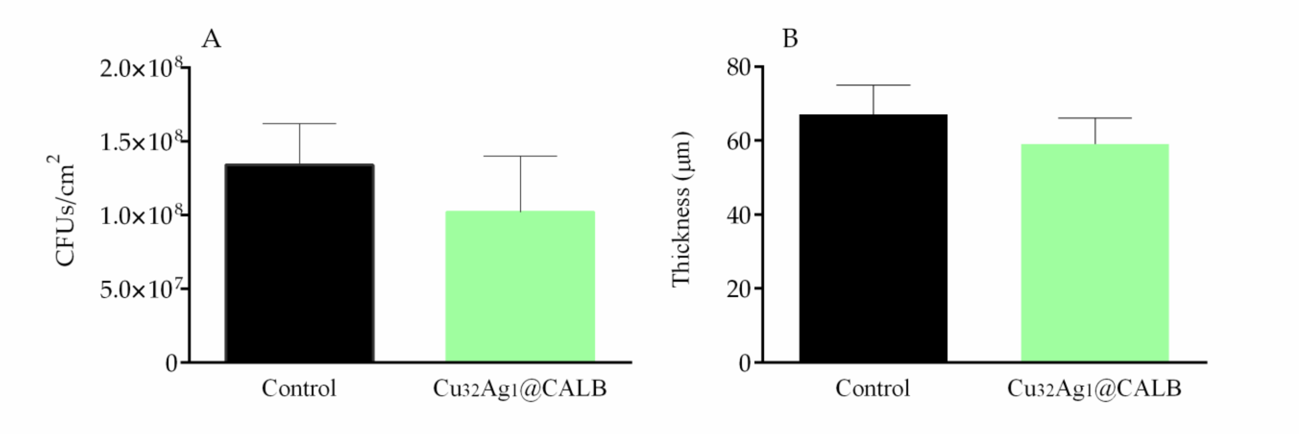

Figure S12. Biofilm culturable cells and biofilm thickness of *Cobetia marina* 3-week-old biofilms developed on polymeric matrix-coated steel coupons (control surface) and on steel coupons functionalized with **$Cu_{32}Ag_1$@CALB**. Mean values and SD from three biological assays, with two technical replicates each, are represented.

F. Metal leaching assessment by Inductively Coupled Plasma-Optical Emission Spectroscopy (ICP-OES)

To evaluate metal leaching, different metal bionanohybrid solutions – **Cu_{33}@CALB**, **$Cu_{32}Ag_1$@CALB**, and **$Cu_{16}Ag_{13}$@CALB**, were freshly prepared in ultrapure water at a final concentration of 250 ppm. In parallel, the bionanohybrid-functionalized surface was also exposed to ultrapure water. Both the bionanohybrid solutions and the functionalized surface were incubated for three days under biofilm formation conditions (25 °C, 185 rpm), corresponding to the period after which the culture medium was replaced during the biofilm formation assays. After incubation, the washing solutions were filtered using 0.22 µm filters (VWR International, Carnaxide, Portugal) and analyzed using an ICP-OES equipped with a nebulizer system and optical emission spectroscopy for detection. To quantify metal content in the samples, a calibration curve was prepared for both copper and silver metals. Measurements were conducted in triplicate, and the results are presented as mean values.

Table S1. Copper (Cu) and silver (Ag) content (ppm) released from bionanohybrids and bionanohybrid-functionalized surface after three days of washing.

Sample	Copper (ppm)	Silver (ppm)
Cu_{33}@CALB	0.020	n.d.
$Cu_{32}Ag_1$@CALB	n.d.	0.030
$Cu_{16}Ag_{13}$@CALB	0.010	0.200
$Cu_{32}Ag_1$@CALB-functionalized surface	0.010	n.d.

n.d. not detected.
